# Cobalt-Catalyzed Methoxycarbonylation of Substituted Dichlorobenzenes as an Example of a Facile Radical Anion Nucleophilic Substitution in Chloroarenes

**DOI:** 10.3390/molecules19055876

**Published:** 2014-05-06

**Authors:** Tatyana S. Khaibulova, Irina A. Boyarskaya, Evgeny Larionov, Vadim P. Boyarskiy

**Affiliations:** 1 Chemistry Department, Saint Petersburg State University, Universitetskiy pr., 26, Staryj Petergof, 198504 St. Petersburg, Russia; 2 Department of Organic Chemistry, University of Geneva, 30 Quai Ernest Ansermet, CH-1211, Geneva 4, Switzerland

**Keywords:** cobalt-catalysed methoxycarbonylation, aryl chlorides, synthesis of substituted benzoic acids, radical anion nucleophilic substitution, *ortho*-effect

## Abstract

A thorough mechanistic study on cobalt-catalysed direct methoxycarbonylation reactions of chlorobenzenes in the presence of methyl oxirane on a wide range of substrates, including poly- and monochloro derivatives with multiple substituents, is reported. The results demonstrate that the reaction is potentially useful as it proceeds under very mild conditions (t = 62 °C, P_CO_ = 1 bar) and converts aryl chlorides to far more valuable products (especially *ortho*-substituted benzoic acids and esters) in high yields. This transformation also offers another opportunity for the utilization of environmentally harmful polychlorinated benzenes and biphenyls (PCBs). This study is the first to discover an unexpected universal positive *ortho*-effect: the proximity of any substituent (including Me, Ph, and MeO groups and halogen atoms) to the reaction centre accelerates the methoxycarbonylation in chlorobenzenes. The effect of the *ortho*-substituents is discussed in detail and explained in terms of a radical anion reaction mechanism. The advantages of the methoxycarbonylation as a model for the mechanistic study of radical anion reactions are also illustrated.

## 1. Introduction

In 1970 Bunnett and Kim [1] proposed a new mechanism for nucleophilic aromatic substitution in non-activated aromatic compounds, which they called radical anion substitution (“unimolecular radical nucleophilic substitution”, S_RN_1) after its key intermediate − a radical anion. In aromatic nucleophilic substitution, this reaction mechanism was the last discovered and still remains far less studied than the others [2]. At the same time, a growing number of examples have become known where reactions proceeding via such a mechanism play an important role in organic synthesis [3,4,5,6] and in the environment [7,8,9,10,11,12,13,14,15]. Therefore, the mechanistic study on radical anion substitution is very timely.

**Scheme 1:**
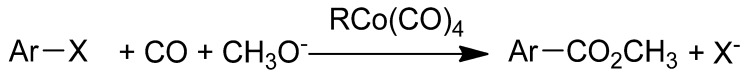
Methoxycarbonylation of aryl halides catalysed with alkyl cobalt carbonyl complex in basic methanol.

The reaction of aryl halide methoxycarbonylation catalysed with alkyl cobalt carbonyl complexes (Scheme 1) provides an interesting model to study the principles of the radical anion transformations in aryl halides [16,17,18,19,20,21,22,23].

Cobalt-catalysed hydroxy- and alkoxycarbonylation of iodo- and bromobenzenes in the system NaH—sodium alcoholate—cobalt acetate (CoCRACO) has been known since 1979 [24,25]. The authors proposed the radical anion mechanism for this reaction. Later it was shown that photochemical initiation can be used in the carbonylation of bromobenzenes instead of a one-electron reducing agent (NaH) [20,26,27,28].

Photochemical carbonylation of substituted chlorobenzenes using cobalt catalysts under low pressure of CO (2 atm) was described in 1986 [29,30,31,32], where the maximum reactivity was noted for substituted chlorobenzoic acids. Unfortunately, photochemical processes have a number of features that make them difficult for preparative use and do not allow them to serve as a full-fledged model for studying the radical anion nucleophilic substitution of aryl halides under dark conditions. Therefore, as a model to study radical anion substitution in aryl chlorides, we have chosen the carbonylation reaction catalysed by anionic alkyl cobalt carbonyl complex (Scheme 1), which occurs under atmospheric pressure and does not require photochemical activation [16,17,18,19,20,21,22,23].

**Scheme 2:**
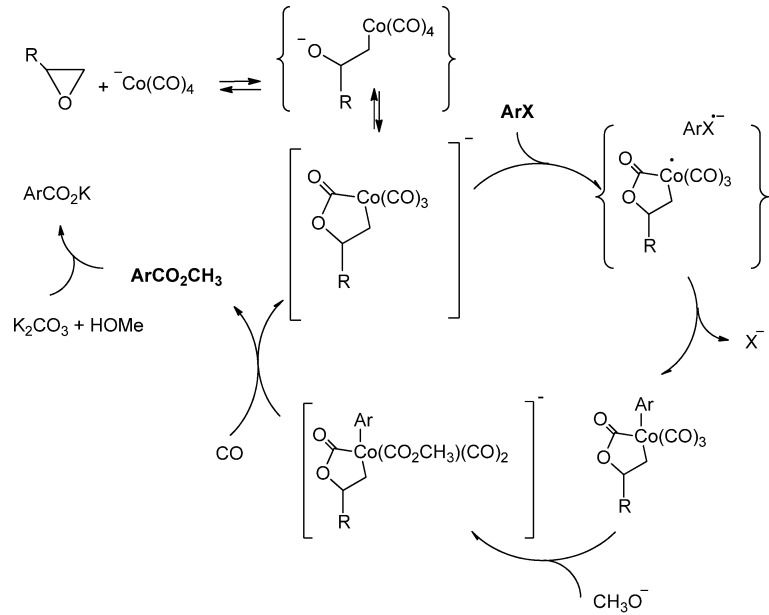
The catalytic cycle of the methoxycarbonylation in presence of methyl oxirane.

Such catalytic systems were first used for the carbonylation of substituted bromobenzenes in 1985 [16]. Originally they were prepared *in situ* from cobalt carbonyl and alkyl halides or dimethyl sulphate [16,17,18,19,20,21]. The main drawback of these systems was a low catalytic activity in carbonylation of substituted chlorobenzenes. Activation of these inert aryl halides was achieved only after the catalyst systems based on cobalt carbonyl and oxirane (or monoalkyl oxiranes) were developed [22,23]. When Co_2_(CO)_8_ is used in presence of methyl oxirane as a catalyst in basic methanol, the methoxycarbonylation proceeds through the formation of an anionic cobalt lactone (Scheme 2) [23,33,34]. It was shown [23,34], that the key step of the process is the radical anion activation of the aryl halide with the anionic cobalt complex. In addition to aryl bromides, some aryl chlorides take part in this reaction, particularly the ones that we discovered in our previous studies: chloronaphthalene [22], PCBs (polychlorinated benzenes) [35], and heteroaryl chlorides [36].

The methoxycarbonylation of only a few dichlorobenzenes has been described under these conditions [37]. At the same time, cobalt-catalysed methoxycarbonylation gives a good opportunity to study the principles of the radical anion processes in chloroarenes. The advantages of this reaction as a model for a mechanistic study in comparison to other radical anion transformations of aryl chlorides are: (i) irreversibility of the methoxycarbonylation process; (ii) simplicity of conversion control; and (iii) the relative ease of the resulting reaction products’ isolation and interpretation of their structures.

Therefore our task was to determine the reactivity of substituted chlorobenzenes in the methoxycarbonylation reaction catalysed by cobalt carbonyl complexes. It has simultaneously pursued two goals: to clarify the reaction’s synthetic possibilities and to use this reaction as a model to study the radical anion substitution in chloroarenes.

## 2. Results and Discussion

First, it was necessary to determine whether chlorobenzene and its derivatives can be carbonylated under the conditions used for the substituted bromobenzenes [22] (in methanol solution at atmospheric pressure of CO and temperature 60–63 °C). Our study showed that unsubstituted chlorobenzene does not react under these conditions. However, the *ortho*- and *meta*-dichlorobenzenes react readily to give the methyl esters of the corresponding chlorobenzoic acids. Therefore diverse substituted dichlorobenzenes were chosen as substrates to study the principles of the methoxycarbonylation reaction.

### 2.1. The Relative Reactivity of Substituted Dichlorobenzenes

**Figure 1:**
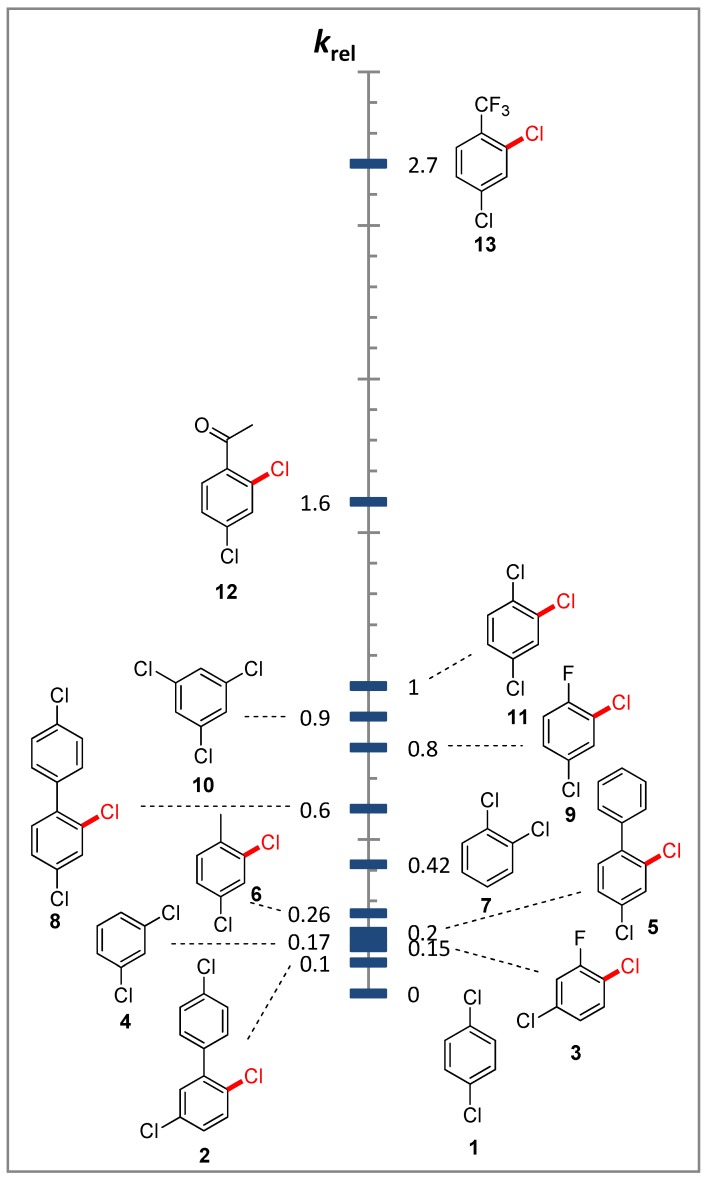
The relative reactivity of substituted dichlorobenzenes (C_6_H_3_Cl_2_X) in the cobalt-catalysed methoxycarbonylation. The C–Cl bond which is preferentially cleaved is marked in red.

We have studied the relative reactivity of several substituted dichlorobenzenes and their derivatives (compounds **1**–**13**, Figure 1) by the competitive reactions method. 

The determination of the relative reaction rates (*k_rel_*) was carried out via measurements of the decreases in the initial substrate concentrations. GC analysis of reaction mixtures before and after methoxycarbonylation was used as an analytical method. Control experiments showed that these aryl halides are consumed in the system only as a result of the methoxycarbonylation reaction. Following data were obtained on the relative reactivity of substituted dichlorobenzenes (Figure 1). 

It is evident that the electron-withdrawing substituents X in *meta*-dichlorobenzenes (2,4-Cl_2_-C_6_H_3_-X) accelerate the methoxycarbonylation reaction: the reaction rate increases in the order H < F < Cl < COCH_3_ < CF_3_. It is not surprising given the nature of the nucleophilic substitution in aryl halides. In addition, it is consistent with our earlier data on the electronic effects of substituents in the methoxycarbonylation of substituted bromobenzenes [23].

At the same time, the presented results (Figure 1) indicate a complex effect of substituents on the reaction rate. For example, the transition from *meta*-dichlorobenzene (**4**) to 2,4-dichlorotoluene (**6**) also accelerates the methoxycarbonylation process, despite the electron-donating nature of the methyl substituent. This indicates the presence of a special substituent effect, which cannot be described only within the concept of the electronic influence. Greater reactivity of *ortho*-dichlorobenzene (**7**) in comparison with *meta*-dichlorobenzene (**4**) (Figure 1) is another fact, which cannot be explained into the frames of the substituents’ electronic influence on the aromatic substrate reactivity in the reaction.

These two contradictions suggest that the determining factor in the radical anion nucleophilic substitution of aryl chlorides is not the nature of the substituents but rather their relative positions. In this regard, the study of the effect of substituents position has become of great importance for the determination of the nature of the substituents’ influence on the aryl chloride reactivity in the anion radical nucleophilic substitution reactions.

### 2.2. Regioselectivity in the Methoxycarbonylation of Substituted Dichlorobenzenes

The data presented above shows only the overall substrate reactivity (without consideration of the substituent position). In order to study the steric effects of substituents, we measured the regioselectivity of the first stage for the methoxycarbonylation of the substituted dichlorobenzenes. The essence of such an approach is to make difference between the chlorine atoms via the introduction of a substituent into the dichlorobenzene molecule. For example, compound **9** has chlorine atom in the *ortho*-position relative to the fluorine atom and the other one in the *para*-position. Determination of the methoxycarbonylation regioselectivity for compound **9** allows us to determine where the influence of fluorine atom is stronger, in the *ortho*-position or in the *para*-position. This method has the advantage of comparing the intersubstrate selectivity definition, since it minimises the influence of extraneous factors that may manifest themselves in other stages of the multistage process.

**Scheme 3:**
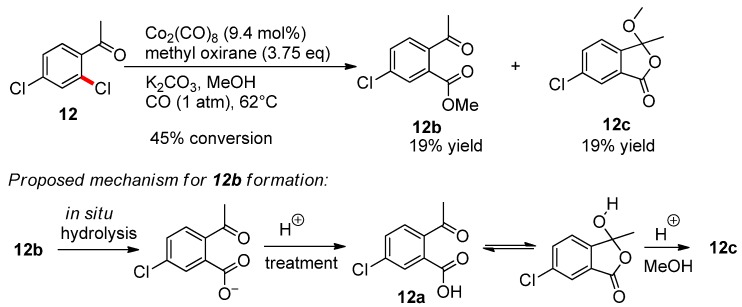
Monocarbonylation of compound **12**.

The methoxycarbonylation of compounds **1**–**15** was carried out up to low conversion (usually 10%–40%) to define the regioselectivity of the first stage. The resulting monocarboxylic acids were isolated (generally as their methyl esters) and their structures were determined by NMR analysis. The only difficulty was to establish the structures of two isomeric products from the monocarbonylation of compound **12** probably formed during the reaction mixture treatment (compounds **12b** and **12c**, Scheme 3). The structure of unexpected product **12c** was unambiguously determined by spectroscopic and spectrometric methods (see Supplementary Information for details). Thus, despite the fact that the reaction in this case yields a mixture of isomers, both obtained products showed a quantitative regioselectivity for the methoxycarbonylation of compound **12** in the position which is *ortho* to the acetyl substituent. Moreover, in all studied compounds **1**–**15**, *ortho*-substituent directs reaction into position 2 exclusively regardless its own nature (see Figure 1, Scheme 3 and Table 1). The regioselectivity study indicates thus an unprecedented universal effect of *ortho*-substituents (including such ones as Me in compound **6** and MeO in compound **14**) observed in reactivity studies.

**Table 1:** The synthesis of the substituted benzoic acids ^a^. 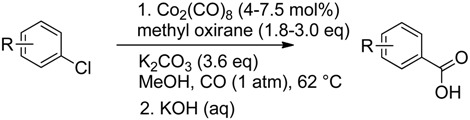

Entry	Substrate	Product	Isolated yield, %
**1**	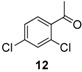	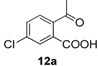	82
**2**	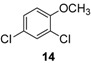	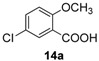	73
**3**	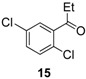	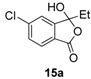	63
**4**	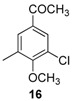	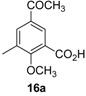	80

^a^ For the reaction conditions see the Experimental Section.

The *ortho*-effect, observed in methoxycarbonylation reaction, is fundamentally different from the effect of *ortho*-substitution described in the literature for reactions which proceed via the classical aromatic nucleophilic substitution mechanism. For example, when methanolysis of substituted chlorobenzene occurs via the S_N_Ar mechanism, the reaction rate for the *ortho*-substituted aryl chlorides is lower than for the corresponding *para*-substituted chlorobenzenes [38,39]. Thus, in accelerating the methoxycarbonylation reaction with an *ortho*-substituent there is a fundamental difference between the steric effect of substituents in the reactions of the radical anion substitution and the same in aromatic nucleophilic substitution reactions (S_N_Ar).

Positive *ortho-*effects in the radical anion substitution reactions have been observed previously by several researchers but only for substituents that have the mesomeric acceptor effect [40,41,42,43,44,45]. For example, there is a positive *ortho*-effect of the acetyl group for radical anion reactions of 2- and 4-chloroacetophenone, manifested in greater dehalogenation rate of 2-chloroacetophenone in comparison with 4-chloroacetophenone in irradiated aqueous solutions [40] and during the reduction with 1,3-dimethyl-2-phenylbenzimidazoline [41]. A similar accelerating effect is demonstrated by *о-*CN [42] and *о-*COPh [41] groups at dehalogenation and *о-*CO_2_Me group in reactions of methyl 2,5-dichlorobenzoate or methyl 3,6-dichloro-2-methoxybenzoate with tin-centred [43,44] and sulphur centred [45] nucleophiles.

Substituents in the *ortho*-position to the reaction center sometimes accelerate interactions of aryl halides with transition metal complexes [46], but these examples refer to substituents that can act as ligands: either heteroatoms with electron lone pairs or groups with multiple bonds. In these cases, the cause of the positive *ortho*-effect is the ability to chelate with the substituents. The attack preference in the *ortho*-position to methyl group observed in the studied methoxycarbonylation cannot thus be explained.

### 2.3. Possible Driving Forces of the Ortho-Effect

**Scheme 4:**
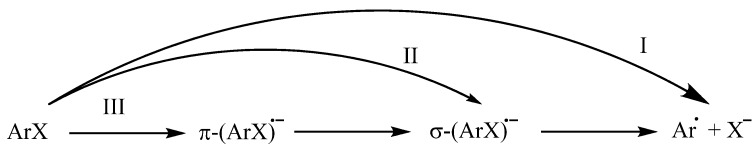
The possible pathways of the C–X breaking for an aryl halide radical anion.

The background for the observed positive *ortho-*effect might be the peculiarities of the mechanism of cobalt-catalysed methoxycarbonylation. As noted earlier, this reaction proceeds via a radical anion mechanism. Carbon-halogen bond breaking can occur concertedly or stepwise [47,48]. If the excitation of the unpaired electron to low-lying vacant MOs and the following internal coordinates rearrangement gives a structure of other geometry than the initial one, π-radical anion → σ-radical anion transfer can be possible. In this case, both structures correspond to different local minima on the potential energy surface of the radical anion ground state. It was shown that the reaction coordinate π-radical anion → σ-radical anion transfer is associated with elongation of the C–X bond and displacement of the halogen atom from the plane of the aromatic ring [49]. That is, in general terms, the scheme of the C-X breaking is as follows (Scheme 4).

Depending on the substrate structure, the process of radical anion activation of the aryl halide proceeds mainly via pathway **I**, **II** or **III**. Aryl chlorides with electron-withdrawing substituents (*viz.* Ac and CN) in the aromatic ring as well as condensed aromatic system contain low-lying π*-orbitals so the radical anion substitution for these substrates takes place mainly along pathway **III** [49]. Aryl bromides and aryl chlorides without acceptor substituents often react via the concerted mechanism **I** [48]. Therefore the influence of the substituent on the reactivity of the aryl halide can be fundamentally different, depending on which of these groups the aryl halide falls into.

A theoretical study on the electronic nature of radical anions for the family of halobenzonitrile and haloacetophenone compounds was carried out by Pierini and Duca [49]. The authors explained positive *ortho*-effect of the acetyl group on the fragmentation rate of haloacetophenone in terms of π* and σ* orbital isomeric radical anions, their energy difference and the probability of an intramolecular electron-transfer reaction from the π* to the σ* system. Santiago and coworkers [44,45] came to the same conclusions about the impact of a methoxycarbonyl group, performing calculations for substituted methyl benzoates. However, it should be noted that the main geometric, thermodynamic and kinetic parameters of radicals and radical anion intermediates were determined in gas phase. This increases the relative role of the radical anion of π-type. 

One way to determine the reaction path is scanning of the radical anion potential energy surface (PES) along the coordinate of the breaking C–Cl bond. We calculated the cross sections of PES for anion radicals of substituted dichlorobenzenes **5**, **12** and **13** at stretching coordinates of both C–Cl bonds in each compound. Scanning the potential energy surface of radical anion along the reaction coordinate was carried out with full optimization of other parameters at each scan step. The PES scan studies have been performed at the B3LYP/6-31+G* level of theory (effects of methanol solvent were accounted within PCM formalism). 

**Figure 2:**
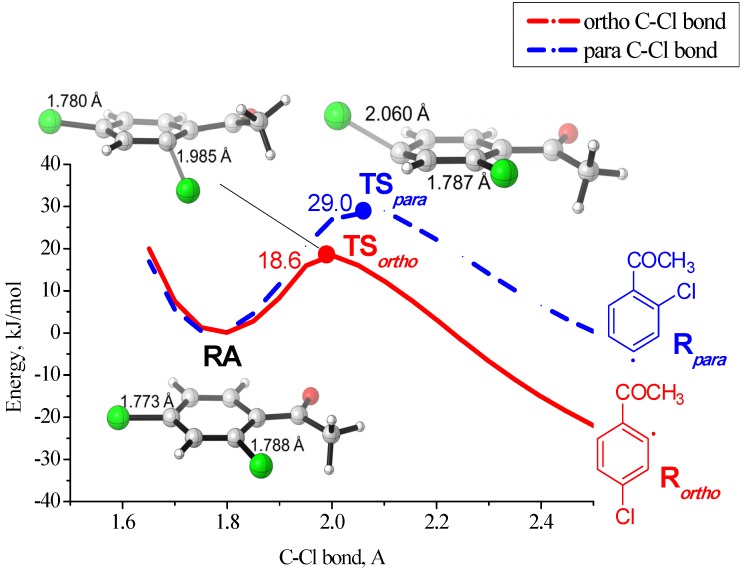
Relaxed potential energy diagram (employing optimised geometry at each point) for the dissociation of chlorides anions from radical anion RA of compound **12**. Structures of radical anion RA and transition states TS*_para_* and TS*_ortho_* with the C–Cl bond distances are shown.

**Scheme 5:**
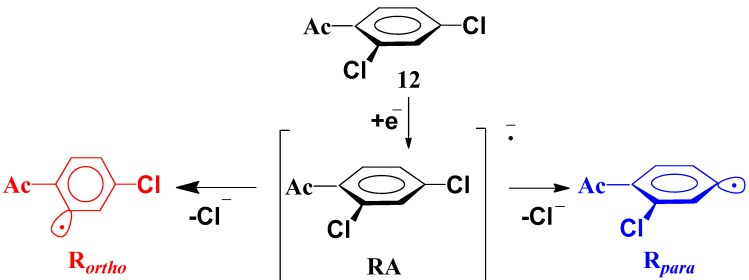
Stepwise fragmentation of the radical anion of the dichlorobenzene **12**.

The computational results for compound **12** are shown in Figure 2 (see Supplementary Information for the energy profiles with other substrates). The electron transfer to a neutral molecule leads initially to the formation of a radical anion **RA** of π-type. It is evident by the presence of a minimum in the potential energy curves. The fragmentation of the radical anion is stepwise and occurs with the activation barrier (Scheme 5). Therefore the reaction takes place along path **III** (Scheme 4). Stabilisation of the π-type radical anion compared to the σ-radical anion in this case is due to the conjugation of the phenyl ring and carbonyl group, leading to partial delocalisation of negative charge and unpaired electron density at the substituent.

The C–Cl bond breaking in *ortho*-position to the substituent has a lower activation barrier (18.6 kJ/mol) compared with C–Cl breaking in para-position (29.0 kJ/mol). This is in a full agreement with the experimentally observed regioselectivity of the methoxycarbonylation reaction (Scheme 3). Notably, the transition state **TS*_para_*** which leads to the *para* C–Cl bond cleavage to give a radical **R*_para_*** is a late TS compared with **TS*_ortho_*** (Figure 2). We obtained similar results by calculating the possible radical anions fragmentation pathways of PCBs, for which the reaction also proceeds by stepwise path **III** [50,51].

For all other computationally studied substrates the ground state of the radical anions formed at the stage of the intermolecular electron transfer are the radical/anion pair. Calculations of such systems are more complicated. All these radical anions do not correspond to minimum on PES. Therefore the decay of the radical anions is barrier-free (pathway **I**). The PE curves along the *ortho-* C–Cl bond coordinates are the lowest for these substrates (see Supplementary Information). It may influence the cleavage of the C–Cl bond at the *ortho*-position with respect to the substituent, which corresponds to the experimental data on the selectivity of the methoxycarbonylation reaction.

Thus, the calculations show that in both cases of the reaction mechanism in a concerted or stepwise process, the cleavage of the C–Cl bond in the *ortho*-position to the substituent is more energetically favourable, regardless electron donating/withdrawing ability of the substituent. This is in a full agreement with the experimentally observed *ortho*-effect of substitutions in the cobalt-catalysed methoxycarbonylation of aryl chlorides.

### 2.4. Synthetic Importance

The feature of cobalt-catalysed aryl chlorides activation was used for the synthesis of substituted chlorobenzoic acids from the corresponding dichlorobenzenes. Basic hydrolysis of the reaction mixture after carbonylation followed by acidification gave several acids in good isolated yields (Table 1). The carbonylation reaction can readily be scaled up to 100 mmol without serious loss of yield (69% *vs.* 73% in case of **14**). For the success of the reaction the substrate must have a substituent in *ortho*-position to one of the chlorine atoms. At this position it has a strong orienting effect, which determines the regioselectivity. Electronic effects of substituents do not play a large role (see product **14a**); although the acceptor substituent is preferable, as it increases the reaction rate and gives complete conversion of substrate after the shorter reaction time. The observed “positive *ortho*-effect” is of a great importance for the methoxycarbonylation reaction used in organic synthesis. It allows the methoxycarbonylation of even substituted chlorobenzenes, which have several alternate donors into the benzene ring, such as compound **16** (Table 1).

One should keep in mind in discussing of the synthetic role of the reaction that nitroaromatic compounds inhibit the cobalt-catalysed methoxycarbonylation (as we have shown previously for aryl bromides) [20,23,26,27,28]. Preparation of alkoxycarbonyl benzoic acids and benzene dicarboxylic acids using the proposed method is also challenging, since most of the esters are hydrolyzed during the process, and the resulting halogen carboxylate anions are inert in the methoxycarbonylation. At the same time, the acyl groups are stable under the reaction conditions and have a strong accelerating effect, which makes the proposed method useful in the synthesis of various substituted acyl benzoic acids.

## 3. Experimental

### 3.1. General Information

Reactions were carried out under argon using a standard manifold with vacuum and argon lines and with magnetic stirring in a jacketed glass reactor for thermostatic experiments equipped with septa or reflux condensers with bubble-counters. All NMR spectra were recorded at ambient temperature on a Bruker DPX 300 spectrometer at 300.13 (^1^H) and 75.03 (^13^C) MHz, respectively. The solvent was CDCl_3_. The NMR spectra are referenced to tetramethylsilane as an internal standard (*δ* = 0 ppm) using the signals of the residual protons of CHCl_3_ (7.26 ppm) in CDCl_3_. Multiplicities of signals are described as follows: s = singlet, br. s = broad singlet, d = doublet, t = triplet, q = quartet, quint = quintet, m = multiplet. Multiplicities in the ^13^C NMR spectra were determined by DEPT measurements. ESI-HRMS were obtained on TOF Micromass LCT spectrometer (Bruker). Elemental analyses were performed in the Organic Analytical Laboratory of St. Petersburg State University Chemistry Department with CHNS elemental analyser Euro EA 3028 – HT. GC system (Chrom-5): PID, the carrier gas—argon (20–30 mL/min). Glass column packed with SE-30 (10%) on Chromaton N-Super (80–100 mesh) as support was used for the analysis, diameter of 3 mm, length 2500 mm. Evaporator temperature 200 °C. The column temperature ranged from 130 °C to 200 °C depending on the studied pairs of substrates. Regular analytical TLC analyses were performed on MERCK ready-to-use plates with silica gel 60 (F_254_). Column chromatography: Merck silica gel, grade 60, 0.040–0.063 mm. Infrared spectra (4000–400 cm^−1^) were recorded on a Shimadzu FTIR-8400S instrument using KBr pellets. Commercially available organic and inorganic reagents (chemically pure grade) were used without further purification. Potassium carbonate (p.a.) was dried at 150 °C for 6 h. Cobalt carbonyl is a commercial reagent (Merck Millipore, Russia). 5-Chloro-4-methoxy-3-methylacetophenone and 2,5-dichloropropiophenone were granted for research by “Vekton Ltd”. (St. Petersburg, Russia). 2,4- and 2,5-Dichlorobiphenyls were synthesised via the Cadogan reaction with the standard procedure [52]. 2,4,4'- and 2,4',5-Trichlorobiphenyls were obtained by chlorination of the corresponding dichlorobiphenyls with the standard procedure [53].

### 3.2. Quantum Chemical Calculations

The quantum chemical calculations were performed using Gaussian 03 software [54] employing the Becke three parameter hybrid exchange in conjunction with the correlation functional developed by Lee, Yang and Parr (B3LYP). The 6-31+G(d) basis set was employed for the structural optimisation in methanol. The open shell model (unrestricted) was used for the anionic radicals and neutral molecules, respectively. The polarisable continuum model has been employed for the solvent. The relaxed based potential energy scanning (full energy optimization at each step) was performed for the radical anions as a function of the C–Cl bond distance in steps of 0.05 Å.

### 3.3. General Procedure for Blank Experiments

K_2_CO_3_ (2.5 g, 18 mmol) and methanol (10 mL) were placed in a jacketed glass reactor. The intensively stirred mixture was subjected to a flow of carbon monoxide, preliminary passed through methanol, at room temperature for 20 min followed by the insertion of Со_2_(СО)_8_ (0.050 g, 0.15 mmol) under a CO atmosphere. After the orange colour disappearance, the reaction mixture was heated to 62 °C, and methyl oxirane (0.35 g, 6 mmol) was added via syringe. The reaction mixture was stirred for 10 min and air was bubbled through the mixture for 5 min at 62 °C to destroy the catalytic complex. After that the flask was stopped with a septum again and a solution of an appropriate amount of the two studied substrates and the corresponding internal standard for GC in methanol (1 mL) was added to the reaction mixture (Solution **A**). The resulting mixture was stirred at 62 °C for 2 h. After that a sample of the reaction mixture (0.2–0.3 mL) was taken, diluted with 1 mL of water and extracted 2 times with hexane. The organic layer was analysed with GC and compared with the sample of solution **A**.

### 3.4. General Procedure I for Competitive Methoxycarbonylation

K_2_CO_3_ (2.5 g, 18 mmol), methanol (10 mL), an appropriate amount of the two studied substrates, and the corresponding internal standard for GC (see below) were placed in a jacketed glass reactor. A sample of the reaction mixture (0.2–0.3 mL) was taken for GC analysis. The intensively stirred mixture was subjected to flow of carbon monoxide, preliminary passed through methanol, at room temperature for 20 min followed by the insertion of Со_2_(СО)_8_ (0.050 g, 0.15 mmol) under CO atmosphere. The orange colour of the mixture disappeared after 5 min, indicating tetracarbonyl cobaltate salt formation according to reaction (1) [55]:

3Со_2_(СО)_8_ + 2K_2_СО_3_ → 4KСо(СО)_4_ + 2CoСО_3_ + 8CO (1)

The reaction mixture was heated to 62 °C, and methyl oxirane (0.35 g, 6 mmol) was added via syringe. The conversion was controlled by measurement of the volume of absorbed carbon monoxide. The reaction was carried out until the combined conversions of the substrates had reached 30%–70%. After that a sample of the reaction mixture (0.2–0.3 mL) was taken. Both samples were diluted each with 1 mL of water and extracted two times with hexane. The organic layers were analysed by GC. The relative rate constants were determined from the ratio of unreacted substrates in the reaction mixture after the carbonylation. Blanks experiments showed that concentrations of the starting aryl halides did not diminished in the absence of methyl oxirane, which indicates the absence of side reactions of the substrates in the reaction mixture. Preservation of the material balance testified to the same.

#### 3.4.1. Competitive Methoxycarbonylation of 1,2-Dichlorobenzene (**7**) and 1,3-Dichlorobenzene (**4**)

The reaction was carried out according to general procedure I with **7** (429 mg, 2.9 mmol), **4** (347 mg, 2.4 mmol) and *tert*-butylbenzene (internal standard, 45 mg, 0.34 mmol). The conversion of the starting compounds was 30% and 13% for **7** and **4**, respectively.

#### 3.4.2. Competitive Methoxycarbonylation of 1,2-Dichlorobenzene (**7**) and 1,4-Dichlorobenzene (**1**)

The reaction was carried out according to general procedure I with 7 (438 mg, 3.0 mmol), **1** (350 mg, 2.4 mmol) and *tert*-butylbenzene (internal standard, 45 mg, 0.34 mmol). The conversion of the starting compounds was 48% and <3% for **7** and **1**, respectively.

#### 3.4.3. Competitive Methoxycarbonylation of 1,2-Dichlorobenzene (**7**) and 1,2,4-Trichlorobenzene (**11**)

The reaction was carried out according to general procedure I with **7 **(560 mg, 4.6 mmol), **11** (843 mg, 3.3 mmol) and 1,2,4,5-tetrametylbenzene (internal standard, 74 mg, 0.55 mmol). The conversion of the starting compounds was 28% and 53% for **7** and **11**, respectively.

#### 3.4.4. Competitive Methoxycarbonylation of 1,2,4-Trichlorobenzene (**11**) and 2,4-Dichloro-1-Fluoro-benzene (**9**)

The reaction was carried out according to general procedure I with **11 **(259 mg, 1.43 mmol), **9** (128 mg, 0.78 mmol), and naphthalene (internal standard, 78 mg, 0.61 mmol). The conversion of the starting compounds was 33% and 28% for **11** and **9**, respectively.

#### 3.4.5. Competitive Methoxycarbonylation of 1,2,4-Trichlorobenzene (**11**) and 1,4-Dichloro-2-Fluoro-benzene (**3**)

The reaction was carried out according to general procedure I with **11** (275 mg, 1.5 mmol), **3** (176 mg, 1.1 mmol) and naphthalene (internal standard, 79 mg, 0.62 mmol). The conversion of the starting compounds was 69% and 26% for **11** and **3**, respectively.

#### 3.4.6. Competitive Methoxycarbonylation of 1,2,4-Trichlorobenzene (**11**) and 1,3,5-Trichlorobenzene (**10**)

The reaction was carried out according to general procedure I with of **11** (208 mg, 1.15 mmol), **10** (206 mg, 1.13 mmol) and naphthalene (internal standard, 54 mg, 0.42 mmol). The conversion of the starting compounds was 45% and 41% for **11** and **10**, respectively.

#### 3.4.7. Competitive Methoxycarbonylation of 1,2,4-Trichlorobenzene (**11**) and 2,4-Dichlorobiphenyl (**5**)

The reaction was carried out according to general procedure I with of **11** (53 mg, 0.3 mmol), **5** (390 mg, 1.75 mmol) and acenaphthene (internal standard, 32 mg, 0.21 mmol). The conversion of the starting compounds was 70% and 23% for **11** and **5**, respectively.

#### 3.4.8. Competitive Methoxycarbonylation of 2,4-Dichlorobiphenyl (**5**) and 2,4,4'-Trichlorobiphenyl (**8**)

The reaction was carried out according to general procedure I with **5** (242 mg, 1.09 mmol), **8** (260 mg, 1.00 mmol) and 1,2-diphenylethane (internal standard, 40 mg, 0.22 mmol). The conversion of the starting compounds was 27% and 61% for **5** and **8**, respectively.

#### 3.4.9. Competitive Methoxycarbonylation of 2,4-Dichlorobiphenyl (**5**) and 2,4',5-Trichlorobiphenyl (**2**)

The reaction was carried out according to general procedure I with of **5** (183 mg, 0.82 mmol), **2** (150 mg, 0.58 mmol) and 1,2-diphenylethane (internal standard, 42 mg, 0.23 mmol). The conversion of the starting compounds was 43% and 25% for **5** and **2**, respectively.

#### 3.4.10. Competitive Methoxycarbonylation of 1,2,4-Trichlorobenzene (**11**) and 2,4-Dichloroaceto-phenone (**12**)

The reaction was carried out according to general procedure I with of **11** (121 mg (0.66 mmol), **12** (401 mg, 2.1 mmol) and naphthalene (internal standard, 49 mg, 0.38 mmol). The conversion of the starting compounds was 25% and 34% for **11** and **12**, respectively.

#### 3.4.11. Competitive methoxycarbonylation of 2,4-Dichlorobiphenyl (**5**) and 2,4-Dichloroaceto-phenone (**12**)

The reaction was carried out according to general procedure I with **5** (497 mg, (2.2 mmol), **12** (142 mg, 0.75 mmol) and acenaphthene (internal standard, 39 mg, 0.25 mmol). The conversion of the starting compounds was 11% and 64% for **5** and **12**, respectively.

#### 3.4.12. Competitive Methoxycarbonylation of 1,3-Dichlorobenzene (**4**) and 2,4-Dichlorotoluene (**6**)

The reaction was carried out according to general procedure I with **4** (308 mg, 2.09 mmol), **6** (434 mg, 2.69 mmol) and *tert*-butylbenzene (internal standard, 42 mg, 0.31 mmol). The conversion of the starting compounds was 44% and 59% for **4** and **6**, respectively.

#### 3.4.13. Competitive Methoxycarbonylation of 1,2,4-Trichlorobenzene (**11**) and 2,4-Dichlorotoluene (**6**)

The reaction was carried out according to general procedure I with **11** (489 mg, 2.69 mmol), **6** (277 mg, 1.68 mmol) and naphthalene (internal standard, 43 mg, 0.34 mmol). The conversion of the starting compounds was 75% and 30% for **11** and **6**, respectively.

#### 3.4.14. Competitive Methoxycarbonylation of 1,2,4-Trichlorobenzene (**11**) and 2,4-Dichloro-1-(trifluoromethyl)benzene (**13**)

The reaction was carried out according to general procedure I with **11**(310 mg, 1.71 mmol), **13** (234 mg, 0.96 mmol) and naphthalene (internal standard, 116 mg, 0.91 mmol). The conversion of the starting compounds was 54% and 87% for **11** and **13**, respectively.

### 3.5. General Procedure II for Determination of Methoxycarbonylation Regioselectivity

K_2_CO_3_ (2.5 g, 18 mmol), methanol (10 mL), and appropriate amount of substrate (see below) were placed in a jacketed glass reactor. The intensively stirred mixture was subjected to flow of carbon monoxide, preliminary passed through methanol, at room temperature for 20 min followed by the insertion of Со_2_(СО)_8_ (0.050 g, 0.15 mmol) under CO atmosphere. The orange colour of the mixture disappeared after 5 min. The reaction mixture was heated to 62 °C, and methyl oxirane (0.35 g, 6 mmol) was added by a syringe. The conversion was controlled by measurement of the absorbed carbon monoxide volume. After reaching of the desired conversion (see below) the reaction mixture was cooled to room temperature and an excess of thionyl chloride (about 0.6 g, 5 mmol) was carefully added into the reaction mixture to pH < 2. The mixture was kept at room temperature for 24 h. A small sample was taken, dissolved with DCM and H_2_O, and the organic layer was analysed by GC to check the conversion. Then the solvent and excess of thionyl chloride were removed from the reaction mixture under reduced pressure. The rest was dissolved with DCM and H_2_O, the organic layer was divided, washed with brine, and dried with Na_2_SO_4_. The solvent was removed under reduced pressure, the resulting mixture was analysed by TLC and ^1^H-NMR methods, and then the methoxycarbonylation products were isolated by column chromatography (eluent—ethyl acetate-hexane, 1:5). The regioselectivity of methoxycarbonylation for compounds **2**, **3**, **5**, **8**, **9** and **11** has been reported by us previously [36,38].

#### 3.5.1. Methoxycarbonylation of 2,4-Dichlorotoluene (**6**)

The reaction was carried out according to general procedure II with **6** (1.5 g, 9.3 mmol) till a 27% conversion. Two liquid fractions were isolated from the reaction mixture in addition to the recovered starting substrate. The first was a mixture of methyl esters of two isomeric chlorotoluenic acids (according to ^1^H-NMR and MS data), total yield 400 mg (23%). The isomers was identified by ^1^H-NMR spectroscopy:

Methyl 5-chloro-2-methylbenzoate (**6a**) [56], *R_f_* *=* 0.54 (ethyl acetate-hexane, 1:5), content in the mixture was 83%. ^1^H-NMR: *δ* = 7.91 (d, *J* = 2 Hz, 1Н), 7.38 (dd, *J* = 2 Hz, *J* = 8 Hz, 1Н), 7.20 (d, *J* = 8 Hz, 1Н), 3.91 (s, 3Н, OCH_3_), 2.58 (s, 3Н, СН_3_) ppm.

Methyl 3-chloro-4-methylbenzoate (**6b**) [57], *R_f_* = 0.52 (ethyl acetate-hexane, 1:5), content in the mixture was 17%. ^1^H-NMR: *δ* = 8.02 (d, *J* = 2 Hz, 1Н), 7.83 (dd, *J* = 2 Hz, *J* = 8 Hz, 1Н), 7.31 (d, *J* = 8 Hz, 1Н), 3.93 (s, 3Н, OCH_3_), 2.44 (s, 3Н, СН_3_) ppm.

The second fraction was dimethyl 4-methylisophthalate (**6c**) [58], *R_f_* *=* 0.42 (ethyl acetate-hexane, 1:5). The yield: 18 mg (1%). ^1^H-NMR: *δ* = 8.59 (d, *J* = 2 Hz, 1Н), 8.06 (dd, *J* = 2 Hz, *J* = 8 Hz, 1Н), 7.35 (d, *J* = 8 Hz, 1Н), 3.95 (s, 3Н, OCH_3_), 3.94 (s, 3Н, OCH_3_), 2.68 (s, 3Н, СН_3_) ppm; ^13^C NMR: *δ* *=* 167.58 (С, *C*O_2_CH_3_), 166.73 (C, *C*O_2_CH_3_), 145.97 (C), 133.03 (CH), 132.37 (CH), 132.29 (CH), 130.15 (C), 128.36 (C), 52.60 (СH_3_, CO_2_*C*H_3_), 52.44 (СH_3_, CO_2_*C*H_3_), 30.10 (C, СH_3_) ppm; MS (ESI): *m*/*z* (positive mode, %) = 209 (100) [*M*+H^+^], 231 (10) [*M*+Na^+^]; HRMS (C_11_H_12_O_4_) 209.0830 (found *M*+H), 209.0814 (calc.), 231.0633 (found *M*+Na), 231.0633 (calc.).

#### 3.5.2. Methoxycarbonylation of 2,4-Dichloroacetophenone (**12**)

The reaction was carried out according to general procedure II with **12** (302 mg, 1.6 mmol) till a 45% conversion. Two methoxycarbonylation products were isolated from the reaction mixture. The first was 6-chloro-3-methoxy-3-methylisobenzofuran-1(3*H*)-one (**12c**), R*_f_* = 0.52 (ethyl acetate-hexane, 1:5). Yield 40 mg (19%), isolated as a white crystals, m.p. 49–50 °C. ^1^H-NMR: *δ* = 7.86 (d, *J* = 2 Hz, 1Н), 7.70 (dd, *J* = 2 Hz, *J* = 8 Hz, 1Н), 7.45 (d, *J* = 8 Hz, 1Н), 3.10 (s, 3Н, ОСН_3_), 1.84 (s, 3Н, СН_3_) ppm; ^13^C-NMR: *δ* *=* 167.0 (C, C-1), 146.1 (C), 137.4 (CH, C-5), 135.3 (C), 129.5 (C), 125.9 (CH, C-7), 124.0 (CH, C-4), 109.1 (C, C-3), 51.8 (СН_3, _OCH3), 25.6 (CH_3_, C-8) ppm; IR (KBr): *v* = 1774 cm^−1^ (C=O); MS (ESI): *m*/*z* (positive mode, %)=213 (5) [*M*+H^+^], 235 (100) [*M*+Na^+^], 251 (15) [*M*+K^+^]; HRMS (ESI): *m*/*z* calcd for C_10_H_9_ClO_3_+H^+^: 213.0318 [*M*+H^+^]; found: 213.0312; calcd for C_10_H_9_ClO_3_+Na^+^: 235.0138 [*M*+Na^+^]; found: 235.0139; calcd for C_10_H_9_ClO_3_+K^+^: 250.9877 [*M*+K^+^]; found: 250.9877; elemental analysis calcd (%) for C_10_H_9_ClO_3_: C 56.49, H 4.27; found: C 56.66, H 4.15.

The second product was methyl 2-acetyl-5-chlorobenzoate (**12b**) [59], R*_f_* = 0.40 (ethyl acetate-hexane, 1:5). Yield 40 mg (19%), isolated as an yellow oil. ^1^H-NMR: *δ* = 7.82 (br. s, 1Н), 7.54 (br. d, *J* = 8 Hz, 1Н), 7.41 (d, *J* = 8 Hz, 1Н), 3.92 (s, 3Н, ОСН_3_), 2.55 (s, 3Н, СН_3_) ppm; ^13^C-NMR: *δ* *=* 201.0 (*С*ОСН_3_), 166. 1 (С, *С*О_2_СН_3_), 140.0 (С, С-5), 136.0 (С, С-2), 131.8 (СН, С-3), 131.5 (С, С-1), 130.5 (СН, С-4), 127.9 (СН, С-6), 52.5 (СН_3_, ОСН_3_), 29.3 (СН_3_) ppm; IR (KBr): *v* =1727 cm^−1^ (C=O in CO_2_Me), 1697 cm^−1^ (C=O); MS (ESI): *m*/*z* (positive mode, %) = 213 [*M*+H^+^] (82), 235 [*M*+Na^+^] (100), 251 [*M*+K^+^] (71); HRMS (ESI): *m*/*z* calcd for C_10_H_9_ClO_3_+H^+^: 213.0318 [*M*+H^+^]; found: 213.0313; calcd for C_10_H_9_ClO_3_+Na^+^: 235.0138 [*M*+Na^+^]; found: 235.0132; calcd for C_10_H_9_ClO_3_+K^+^: 250.9877 [*M*+K^+^]; found: 250.9881.

For additional identification **12b** was saponified with boiling solution of КОН (0.4 g) in a methanol/water mixture (1:1, 20 mL) for 3 h. Methanol was removed, water (5 mL) was added and the reaction mixture was acidified by HCl till *р*Н < 1. The precipitate of 2-acetyl-5-chlorobenzoic acid (**12a**) [60] (10 mg) was filtered off and dried; m.p. 121–122 °С; ^1^H-NMR: *δ* = 7.81 (d, *J* = 2 Hz 1H), 7.64 (br. d, *J* = 8 Hz, 1Н), 7.48 (d, *J* = 8 Hz, 1Н), 2.00 (s, 3Н, СН_3_) ppm.

#### 3.5.3. Methoxycarbonylation of 2,4-Dichloro-1-(Trifluoromethyl)Benzene (**13**)

The reaction was carried out according to general procedure II with **13** (2.0 g, 9.3 mmol) till a 10% conversion. The methoxycarbonylation product was methyl 5-chloro-2-(trifluoromethyl)benzoate (**13a**) [61], R*_f_* =0.52 (ethyl acetate-hexane, 1:5). Yield 200 mg (8.8%), isolated as a colorless oil. ^1^H-NMR: *δ* = 7.79 (d, *J* = 2 Hz, 1Н), 7.70 (d, *J* = 8 Hz, 1Н), 7.58 (dd, *J* = 2 Hz, *J* = 8 Hz, 1Н), 3.96 (s, 3Н, OCH_3_) ppm; ^13^C-NMR: *δ =* 166.2 (*C*O_2_CH_3_), 138.6 (C, С-5), 132.4 (q, *J^2^_С-F_* *=* 94 Гц, С, С-2), 131.6 (СН), 130.9 (СН), 128.6 (q, *J^3^_С-F_* *=* 5 Hz, СН, С-3), 127.5 (С), 123.4 (q, *J^1^_С-F_* *=* 273 Hz, С, СF_3_), 53.5 (ОСН_3_) ppm; MS (ESI): *m*/*z* (positive mode, %) = 239 (58) [*M*+H^+^], 261 (100) [*M*+Na^+^]; HRMS (C_8_H_6_F_3_ClO_4_) 239.0082 (found *M*+H), 239.0081 (calc.), 260.9890 (found *M*+Na), 260.9901 (calc.).

### 3.6. Preparation of Substituted Benzoic Acids

#### 3.6.1. Methoxycarbonylation of 2,4-Dichloroacetophenone (**12**) to Acid **12a**

The reaction was carried out according to general procedure II with **12** (680 mg, 3.6 mmol) for 2 h to reach the full conversion. After absorption of CO had finished, H_2_O (2 mL) was added to the reaction flask and the mixture was stirred at 62 °С for 3 h. Methanol was removed, water (5 mL) was added and the reaction mixture was acidified with HCl to a рН < 1. The precipitate of 2-acetyl-5-chlorobenzoic acid (**12a**) [60] (586 mg, 82%) was filtered off and dried; m.p. 120–121 °С.

#### 3.6.2. Methoxycarbonylation of 2,4-Dichloroanisole (**14**)

The reaction was carried out according to general procedure II with **14** (350 mg, 2.0 mmol) for 4 h till an 80% conversion. After absorption of CO had finished, H_2_O (2 mL) was added to the reaction flask and the mixture was stirred at 62 °С for 3 h. The resulting mixture was cooled and dissolved with DCM (20 mL) and H_2_O (20 mL). The aqueous layer was divided, acidified with aq. HCl to pH = 3–4, and extracted with DCM. The white precipitate formed was filtered off and dried. The resulting product was 5-chloro-2-methoxybenzoic acid (**14a**) [62], isolated as a colorless oil, which solidified slowly. Yield 270 mg (73%). ^1^H-NMR: *δ* = 8.17 (d, *J* = 3 Hz, 1Н), 7.55 (dd, *J* = 3 Hz, *J* = 9 Hz, 1Н), 7.03 (d, *J* = 9 Hz, 1Н), 4.10 (s, 3Н, OСН_3_) ppm. 

#### 3.6.3. Methoxycarbonylation of 2,5-Dichloropropiophenone (**15**)

The reaction was carried out according to general procedure II with **15** (700 mg, 3.5 mmol) for 2 h to reach the full conversion. After absorption of CO had finished, H_2_O (2 mL) was added to the reaction flask and the mixture was stirred at 62 °С for 3 h. The resulting mixture was cooled and dissolved with DCM (20 mL) and H_2_O (20 mL). The aqueous layer was divided and acidified with aq. HCl to pH=3−4. The white precipitate formed was filtered off and dried. The resulting product was 5-chloro-3-ethyl-3-hydroxyisobenzofuran-1(3*H*)-one (**15a**). Yield 460 mg (63%), m.p. 107−108 °C. ^1^H-NMR: *δ* = 7.73 (d, *J* = 9 Hz, 1Н), 7.54–7.51 (m, 2Н), 5.54 (br. s, 1Н, ОН), 2.21–2.10 (m, 2Н, СН_2_), 0.89 (t, *J* = 8 Hz, 3Н, СН_3_) ppm; ^13^C-NMR: *δ* *=* 168.5 (С, С=О), 150.6 (С), 141.8 (С), 131.6 (СН), 127.1 (СН), 125.6 (С), 123.3 (СН), 108.3 (С), 32.3 (СН_2_), 8.1 (СН_3_) ppm; MS (ESI): *m*/*z* (positive mode, %) = 213 (26) [*M*+H^+^], 231 (77) [*M*+Na^+^], 251 (100) [*M*+K^+^]; HRMS (ESI): *m*/*z* calcd for C_10_H_9_ClO_3_+H^+^: 213.0318 [*M*+H^+^]; found: 213.0357; calcd for C_10_H_9_ClO_3_+Na^+^: 235.0138 [*M*+Na^+^]; found: 235.0171; calcd for C_10_H_9_ClO_3_+K^+^: 250.9877 [*M*+K^+^]; found: 250.9897; elemental analysis calcd (%) for C_10_H_9_ClO_3_: C 56.49, H 4.27; found: C 56.42, H 4.17.

#### 3.6.4. Methoxycarbonylation of 5-Chloro-4-Methoxy-3-Methylacetophenone (**16**)

The reaction was carried out according to general procedure II with **16** (480 mg, 2.4 mmol) for 2 h to reach the full conversion. After absorption of CO had finished, H_2_O (2 mL) was added to the reaction flask and the mixture was stirred at 62 °С for 3 h. The resulting mixture was cooled and dissolved with DCM (20 mL) and H_2_O (20 mL). The aqueous layer was divided and acidified with aq. HCl to pH = 3–4. The white precipitate formed was filtered off and dried. The resulting product was5-acetyl-2-methoxy-3-methylbenzoic acid (**16a**). Yield 400 mg (80%), m.p. 122−123 °C. ^1^H-NMR: *δ* = 8.53 (d, *J* = 2 Hz, 1Н), 8.09 (d, *J* = 2 Hz, 1Н), 4.00 (s, 3Н, OСН_3_), 2.65 (s, 3Н, СН_3_), 2.45 (s, 3Н, СН_3_) ppm; ^13^C-NMR: *δ* *=* 197.0 (С, СО), 168.2 (C), 162.5 (C), 136.4 (С), 133.6 (СН), 133.4 (СН), 131.8 (С), 123.0 (С), 62.6 (СН_3_, СО_2_*С*Н_3_), 27.0 (СН_3_, СО*С*Н_3_), 16.7 (СН_3_) ppm; MS (ESI): *m*/*z* (positive mode, %) = 209 (100) [*M*+H^+^], 231 (13) [*M*+Na^+^], 247 (29) [*M*+K^+^]; HRMS (ESI): *m*/*z* calcd for C_11_H_12_O_4_+H^+^: 209.0814 [*M*+H^+^]; found: 209.0808; calcd for C_11_H_12_O_4_+Na^+^: 231.0633 [*M*+Na^+^]; found: 231.0628; calcd for C_11_H_12_O_4_+K^+^: 247.0373 [*M*+K^+^]; found: 247.0369; elemental analysis calcd (%) for C_11_H_12_O_4_: C 63.45, H 5.81; found: C 63.36, H 5.70.

#### 3.6.5. Scaled Procedure for Preparative Reaction

K_2_CO_3_ (50 g, 0.36 mol), methanol (250 mL), and **14** (17.7 g, 0.10 mol) were placed in a three-neck round bottom flask equipped with an inner thermometer, a gas inlet, a rubber stopper, and a magnetic stirring bar. The intensively stirred mixture was subjected to a flow of carbon monoxide, first passed through methanol (the rubber stopper was changed to a bubble counter for this time), at room temperature for 20 min followed by the insertion of Со_2_(СО)_8_ (1.4 g, 0.004 mol) under CO atmosphere. The orange colour of the mixture disappeared after 5 min. The reaction mixture was heated to 62 °C (inner temperature), and methyl oxirane (8.0 g, 0.14 mol) was added via syringe. After several minutes the temperature increased to 63–64 °C, and the reaction proceeded at this temperature. The conversion was controlled by measurement of the absorbed carbon monoxide volume. After 2 h the reaction decelerated strongly, and an additional portion of methyl oxirane (4.0 g, 0.07 mol) was added via syringe. When the absorption of CO was stopped the reaction mixture was cooled to 50–55 °C and 15% aq. KOH (30 mL) was added to the reaction mixture. The mixture was refluxed until the formed ester was hydrolysed completely (TLC control, the rate is depended of the ester structure strongly). Then methanol was removed from the reaction mixture under reduced pressure. The rest was dissolved with H_2_O, filtered and acidified with aq. HCl. The precipitate of **14a** was filtered off, washed with H_2_O, crystallised with aq. EtOH and dried. Yield 12.9 g (69%).

## 4. Conclusions

The cobalt-catalysed methoxycarbonylation of aryl chlorides is potentially very interesting from a synthetic point of view, as well as theoretically. It was found that a variety of polychlorinated benzenes and some substituted monochlorobenzenes with multiple subsituents undergo methoxycarbonylation reactions under very mild conditions (t = 62 °C, P_CO_ = 1 atm). Experiments showed that any substituents in the *ortho*-position to the reactive chlorine atom strongly accelerate this reaction, which proceeds via the radical anion nucleophilic substitution mechanism. In accelerating of a reaction with an *ortho*-substituent there is a fundamental difference between the steric effect of substituents in the reactions of the radical anion substitution and the same in aromatic nucleophilic substitution reactions (S_N_Ar). The quantum chemical calculation confirmed that the cleavage of the C-Cl bond in *ortho*-position to the substituent in the benzene ring is more energetically favourable in radical anion processes. This is one of the driving forces for the unusual universal *ortho*-effect of substitutions in the cobalt-catalysed methoxycarbonylation of aryl chlorides. This effect can be a background for a synthetically useful method for the preparation of *ortho-*substituted benzoic acids starting from easily available aryl chlorides.

## Supplementary Materials

Supplementary materials can be accessed at: http://www.mdpi.com/1420-3049/19/5/5876/s1.
