# Supplementary material for: Cobalt-Catalyzed Methoxycarbonylation of Substituted Dichlorobenzenes as an Example of a Facile Radical Anion Nucleophilic Substitution in Chloroarenes
Source: Molecules. 2014 May 6;19(5):5876–97. doi: 10.3390/molecules19055876 (PMC6270984; doi:10.3390/molecules19055876)
Supplement: Supplementary file 1 [file molecules-19-05876-s001.pdf]

Supplementary Materials

1. Determination of the Structure of Compounds 12b and 12c

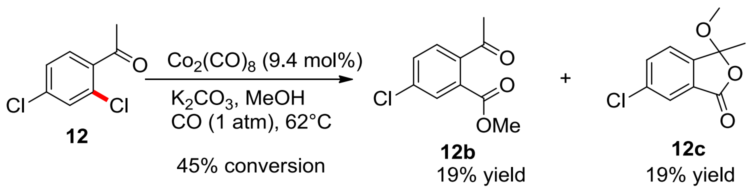

Mass spectrometry data showed that these products were two isomers with the molecular formula $C_{10}H_9ClO_3$ (see Experimental Section). The ^{1}H-NMR spectra of both products were similar and each contained signals of methyl and methoxy groups protons. From the data it can be assumed that these were methyl esters of isomeric acetyl chlorobenzoic acids. At the same time, the ^{13}C-NMR spectra of the compounds were different. The ^{13}C-NMR spectrum of product **12c** did not contain a signal corresponding to the carbonyl carbon atom of the acetyl group, but there was a signal with chemical shift δ = 109.1 ppm, which cannot be attributed to any alleged atom of the methyl esters of acetyl chlorobenzoic acids. The data suggest that the reaction product was the isomer of the ester. Namely, it was 6-chloro-3-methoxy-3-methylisobenzofuran-1(3*H*)-one (compound **12c**) formed during the treatment of the reaction mixture after the carbonylation.

To confirm this assumption, we recorded the infrared spectrum of the product. The absorption band at 1774 cm^{-1} was registered, which corresponds to the stretching vibrations of the γ-lactone carbonyl group. It finally corroborated the proposed structure of compound **12c** and suggested that this product was formed via methoxycarbonylation of a Cl atom in *ortho*-position to the acetyl group.

At the same time, a signal with a chemical shift δ = 201.4 ppm corresponding to the carbonyl carbon of the acetyl group presented in the ^{13}C-NMR spectrum of product **12b**. Also, all other signals corresponded to methyl acetylchlorobenzoate. The presence of a CO_2CH_3 group was confirmed with IR spectroscopy. The absorption bands at 1727 cm^{-1} and at 1697 cm^{-1} are characteristic to the stretching vibrations of the carbonyl group in esters and the carbonyl group in aryl ketones, respectively. Thus, product **12b** was indeed a methyl acetylchlorobenzoate. The position of the methoxy group in product **12b** was determined using ^{13}C-NMR with specific decoupling of the aromatic ring protons (Figure S1).

Figure S1. The signal of carboxyl carbon atom in ^{13}C-NMR spectra of product 12b with specific proton decoupling (**a**)—decoupled of proton H_a; (**b**)—decoupled of proton H_b; (**c**)—decoupled of proton H_c) and without any decoupling (**d**).

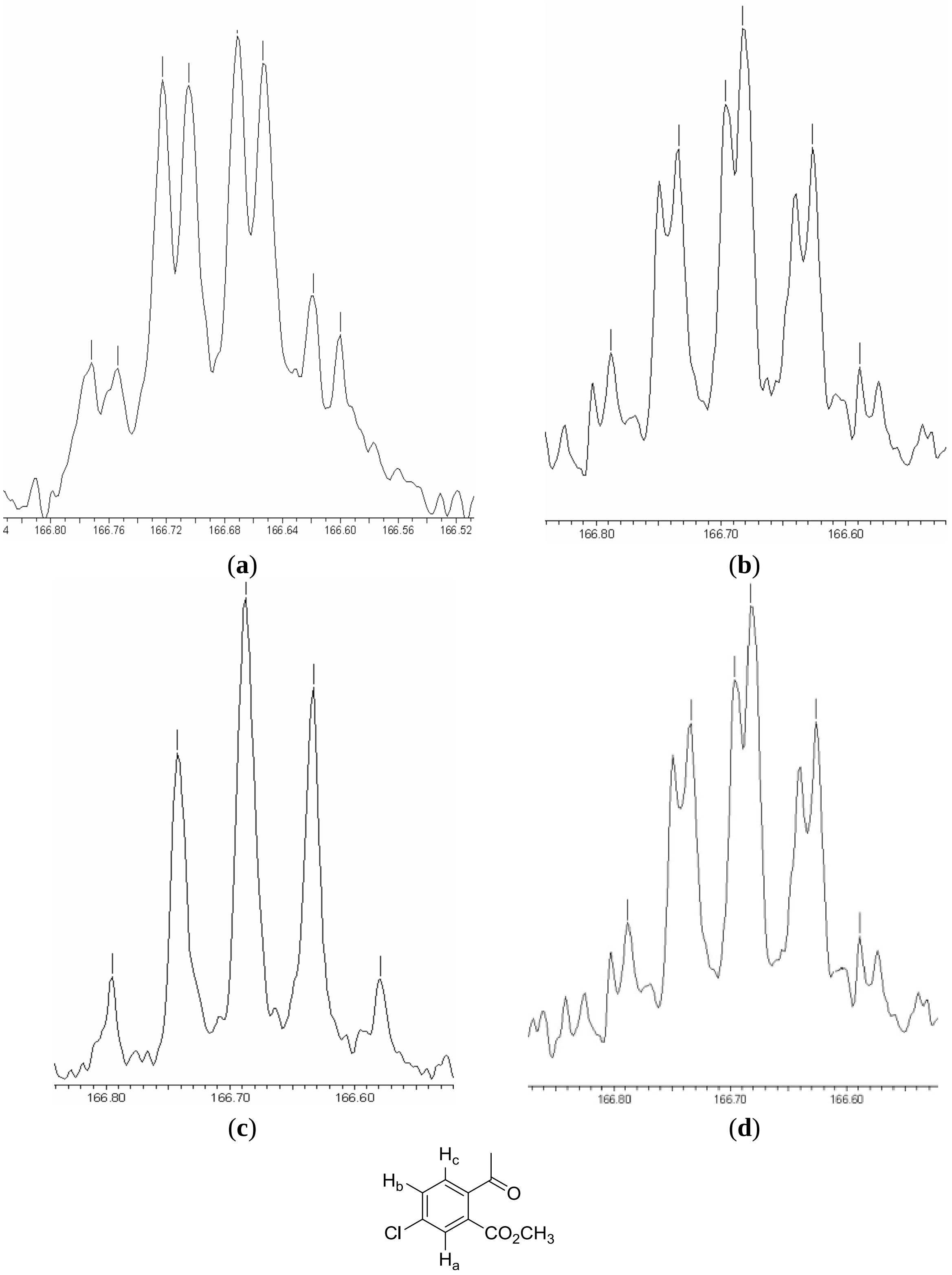

It is evident from the presented data that the interaction of the methoxy carbonyl group with proton $\mathbf{H_a}$ is the strongest (the largest coupling constant disappeared at decoupling, Figure S1a). The C-H coupling constant for proton $\mathbf{H_c}$ has a lower value, and the interaction with proton $\mathbf{H_b}$ is almost absent.

This means that the second product of compound **12** methoxycarbonylation is methyl 2-acetyl-5-chlorobenzoate compound **12b**.

2. NMR Spectra of Products

6c

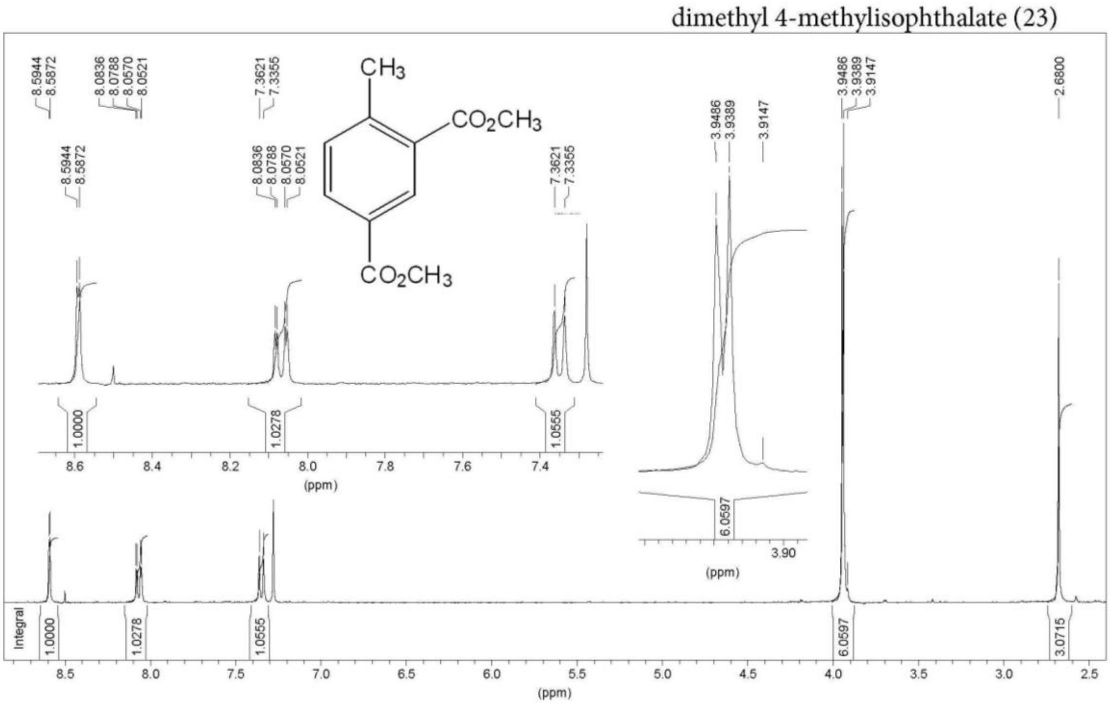

6c

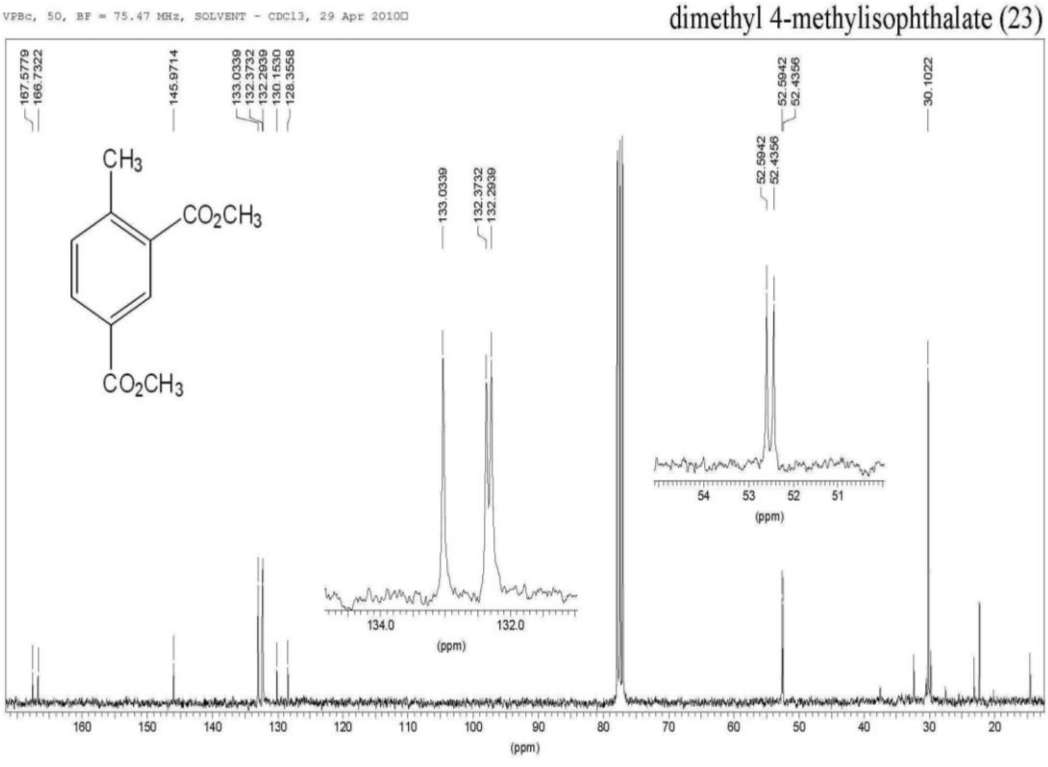

12b

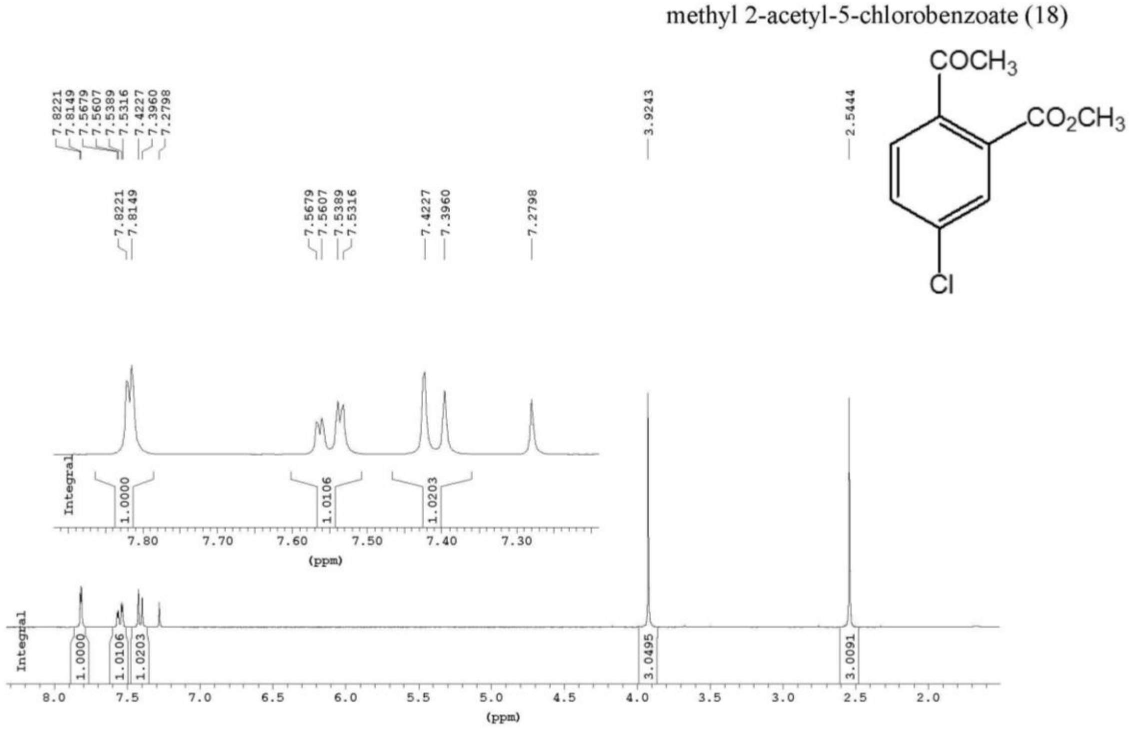
methyl 2-acetyl-5-chlorobenzoate (18)
COCH3
CO2CH3
Cl
7.8221
7.8149
7.5679
7.5607
7.5389
7.5316
7.4227
7.3960
7.2798
3.9243
2.5444
Integral
1.0000
1.0106
1.0203
3.0495
3.0091
(ppm)

12b

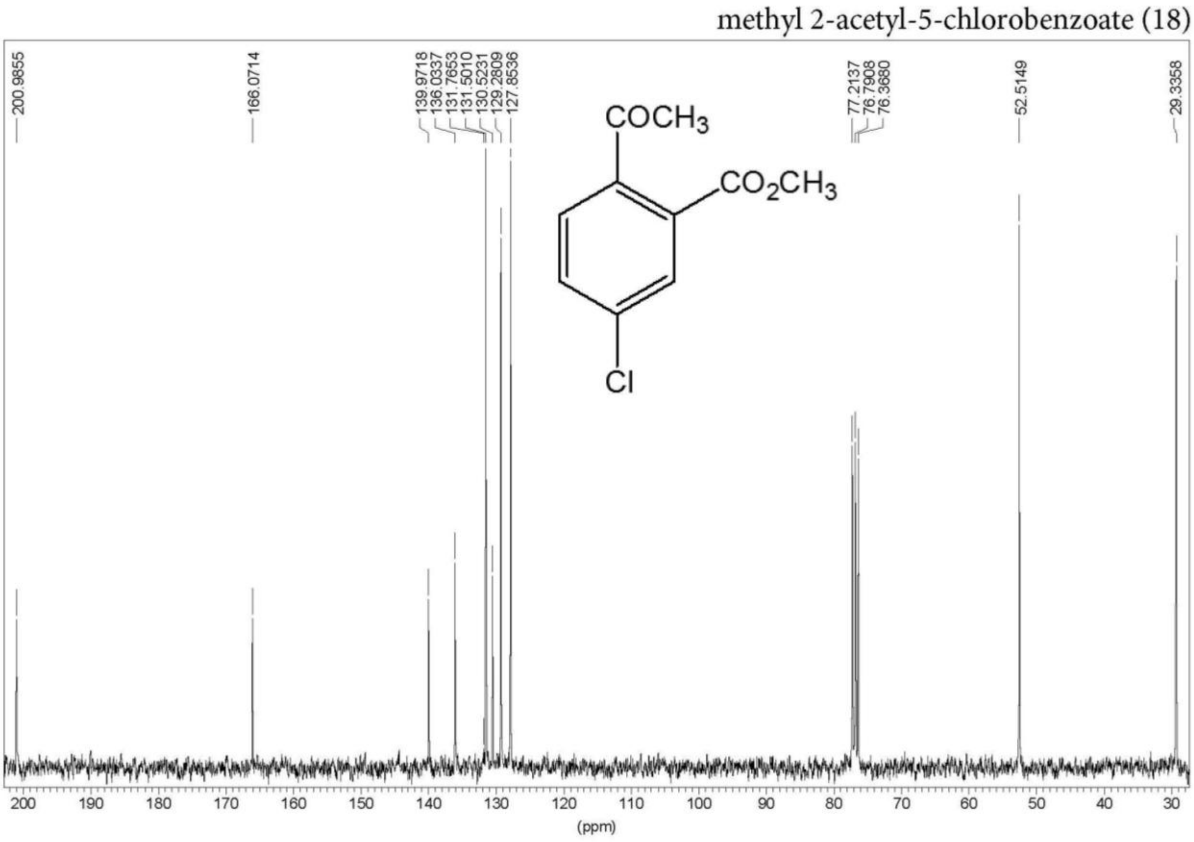
methyl 2-acetyl-5-chlorobenzoate (18)
200.9855
166.0714
139.9718
136.0337
131.7653
131.5010
130.5231
129.2809
127.8536
77.2137
76.7908
76.3680
52.5149
29.3358
COCH3
CO2CH3
Cl
(ppm)

6-chloro-3-methoxy-3-methylisobenzofuran-1(3*H*)-one (**12c**)

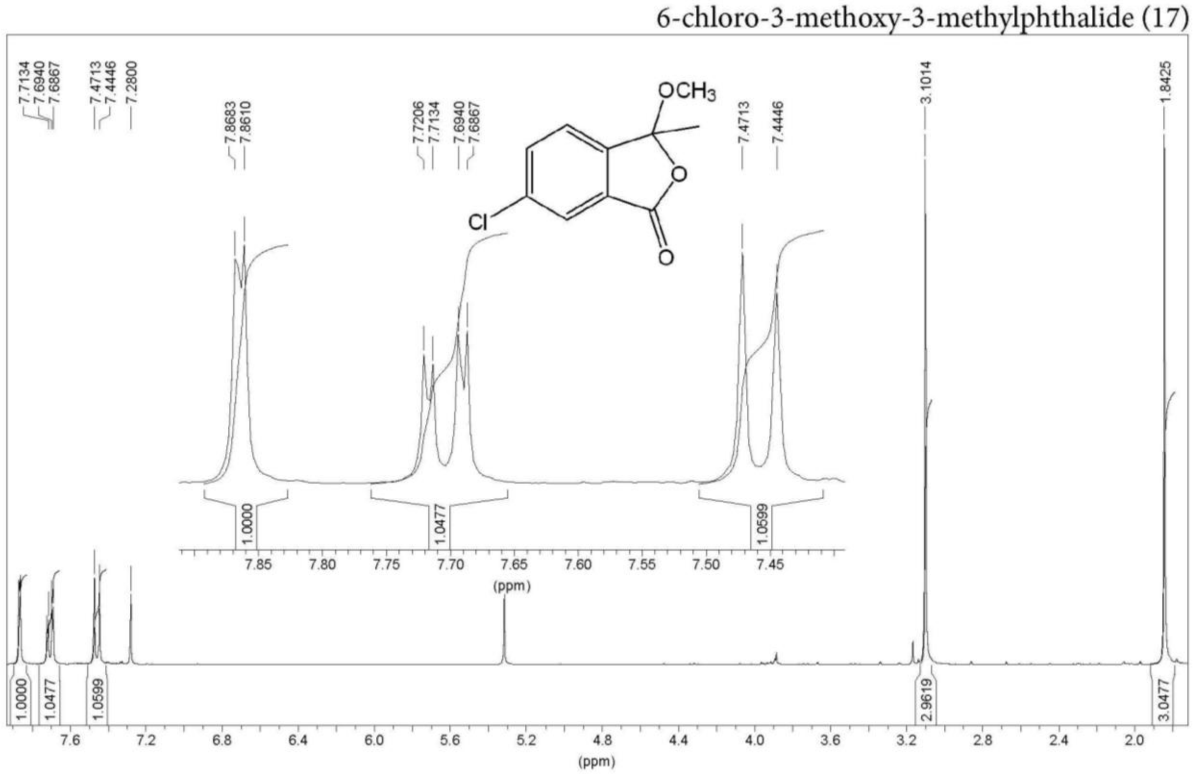

6-chloro-3-methoxy-3-methylisobenzofuran-1(3*H*)-one (**12c**)

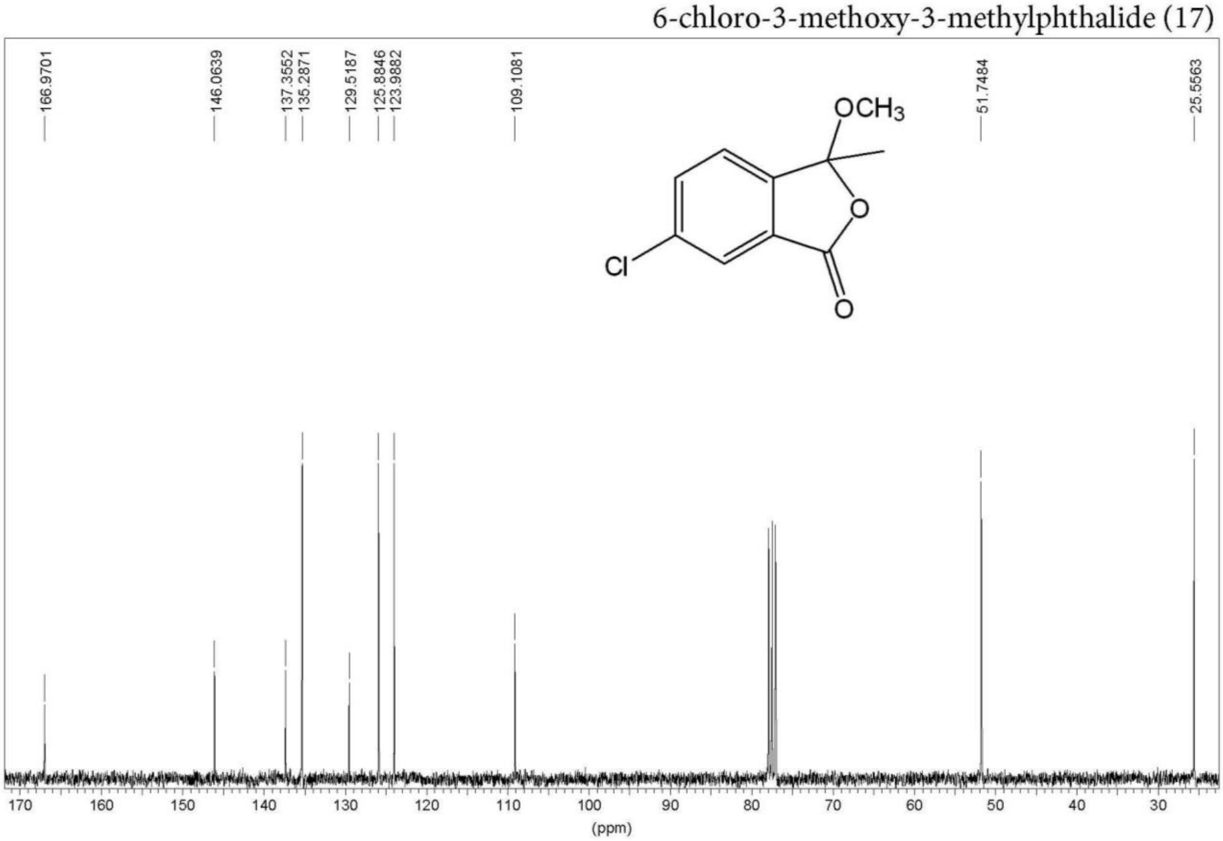

13a

methyl 5-chloro-2-(trifluoromethyl)benzoate (24)

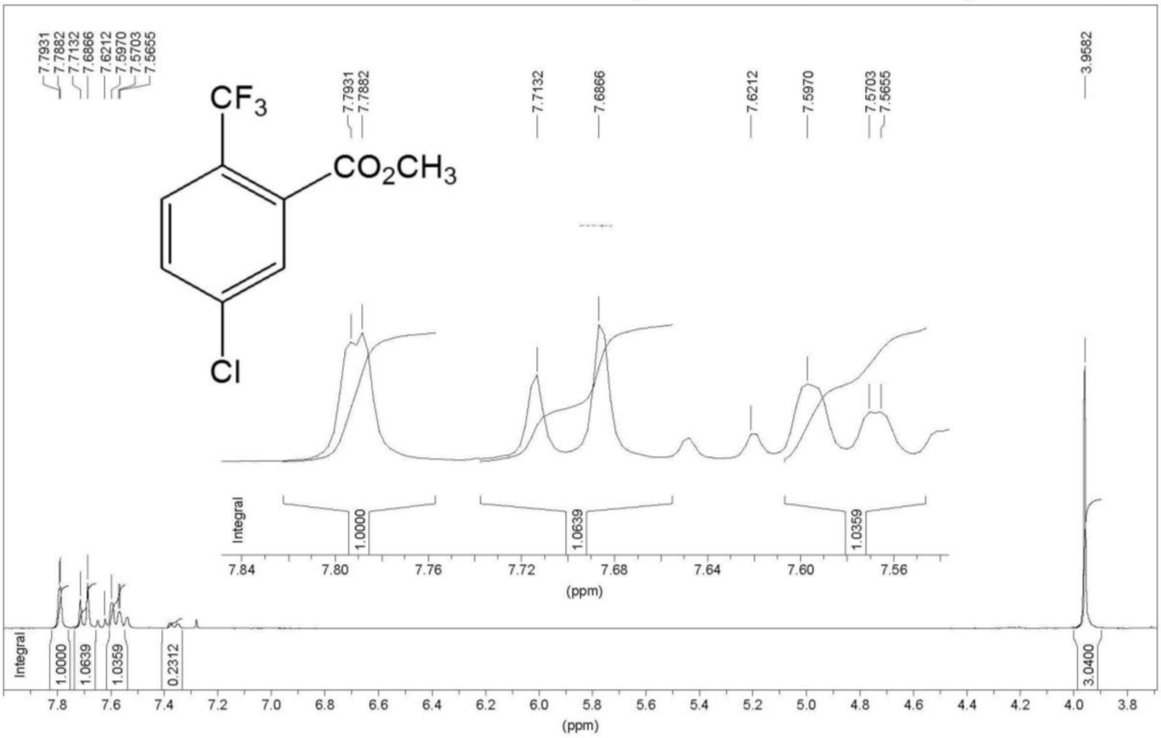

13a

methyl 5-chloro-2-(trifluoromethyl)benzoate (24)

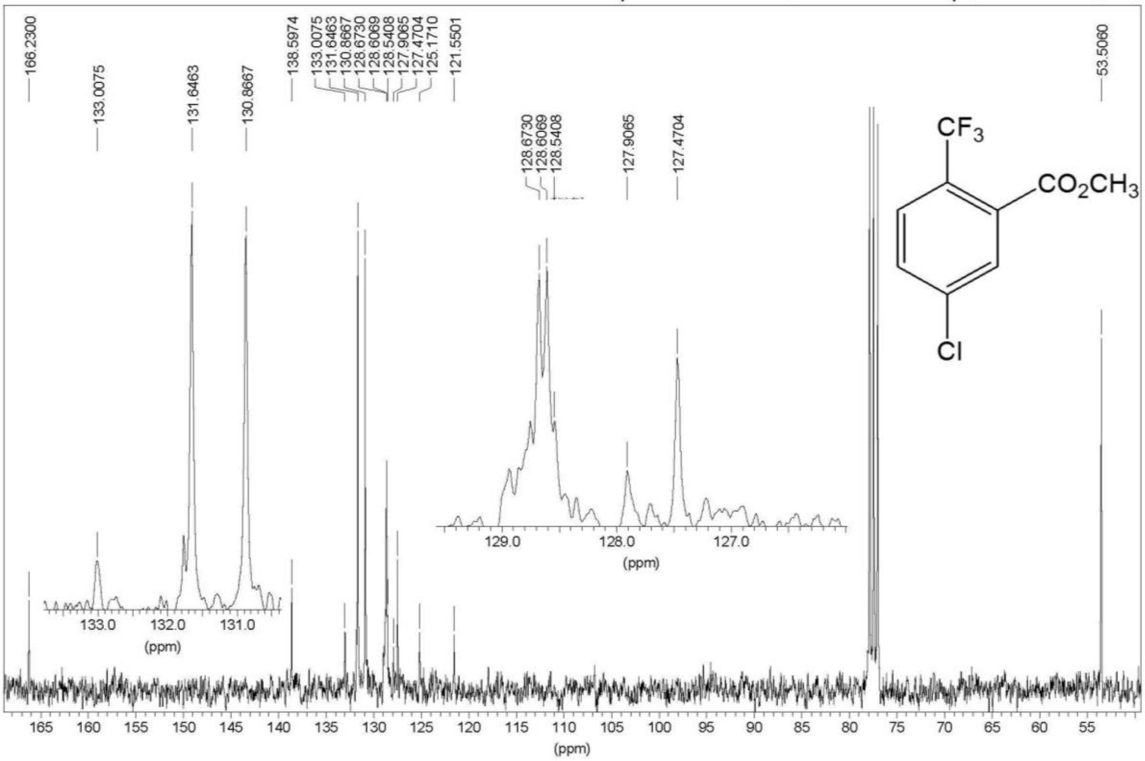

5-chloro-3-ethyl-3-hydroxyisobenzofuran-1(3H)-one (**15a**)

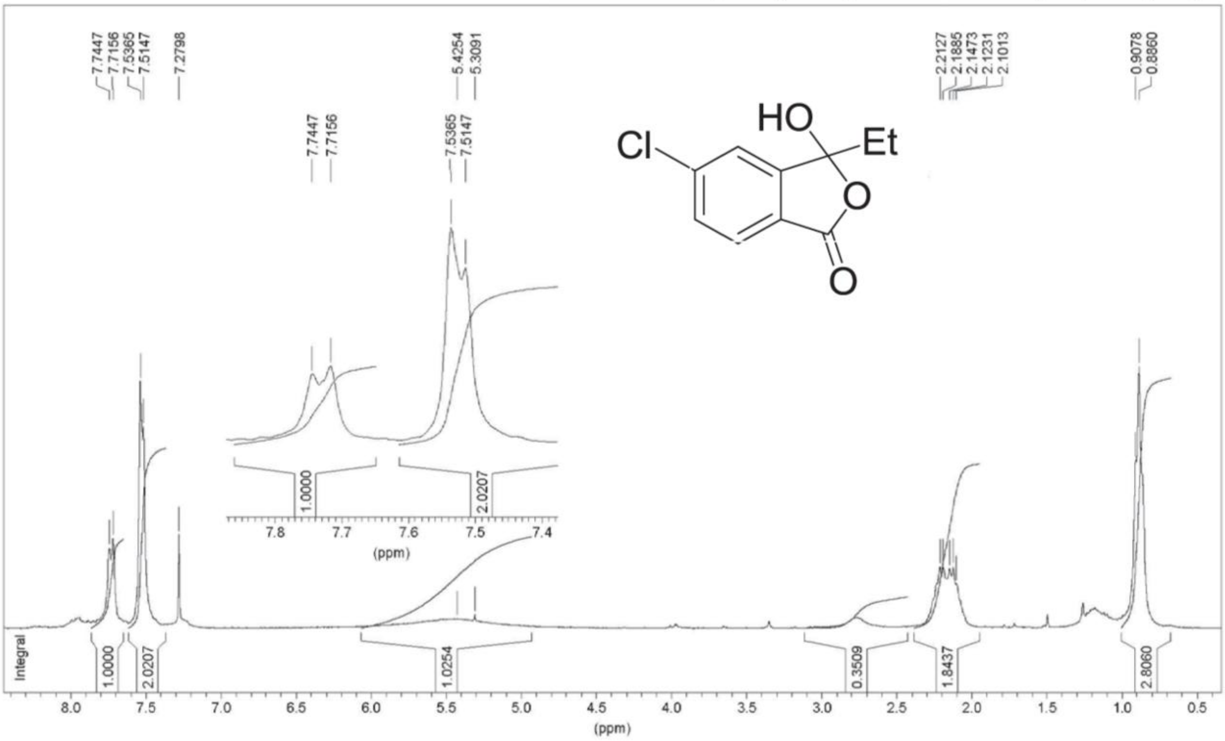

5-chloro-3-ethyl-3-hydroxyisobenzofuran-1(3H)-one (**15a**)

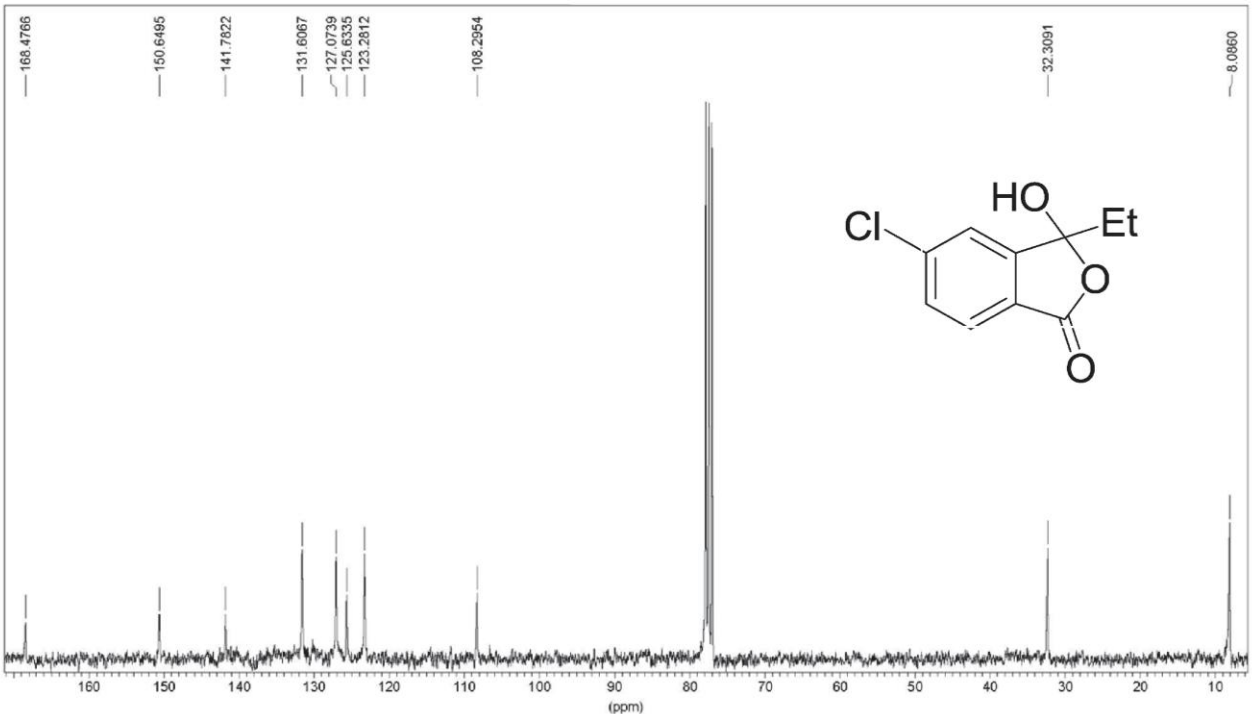

16a

5-acetyl-2-methoxy-3-methylbenzoic acid (20)

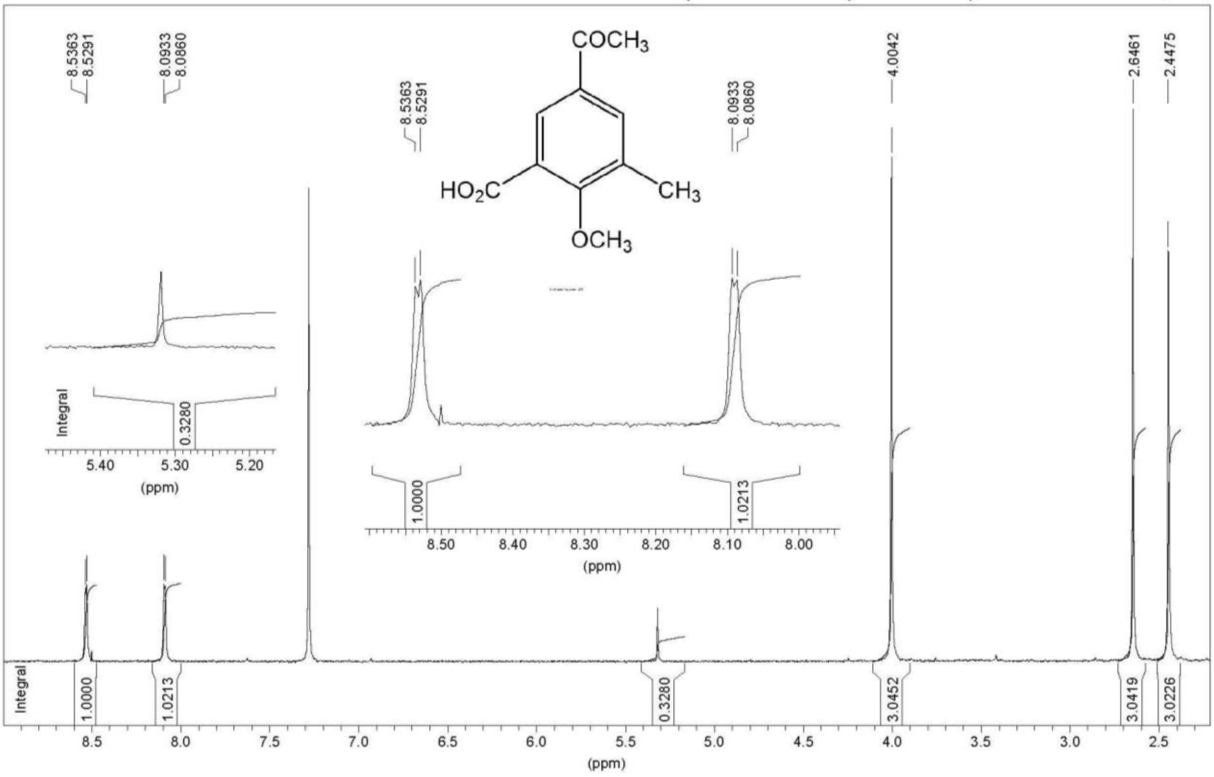

16a

5-acetyl-2-methoxy-3-methylbenzoic acid (20)

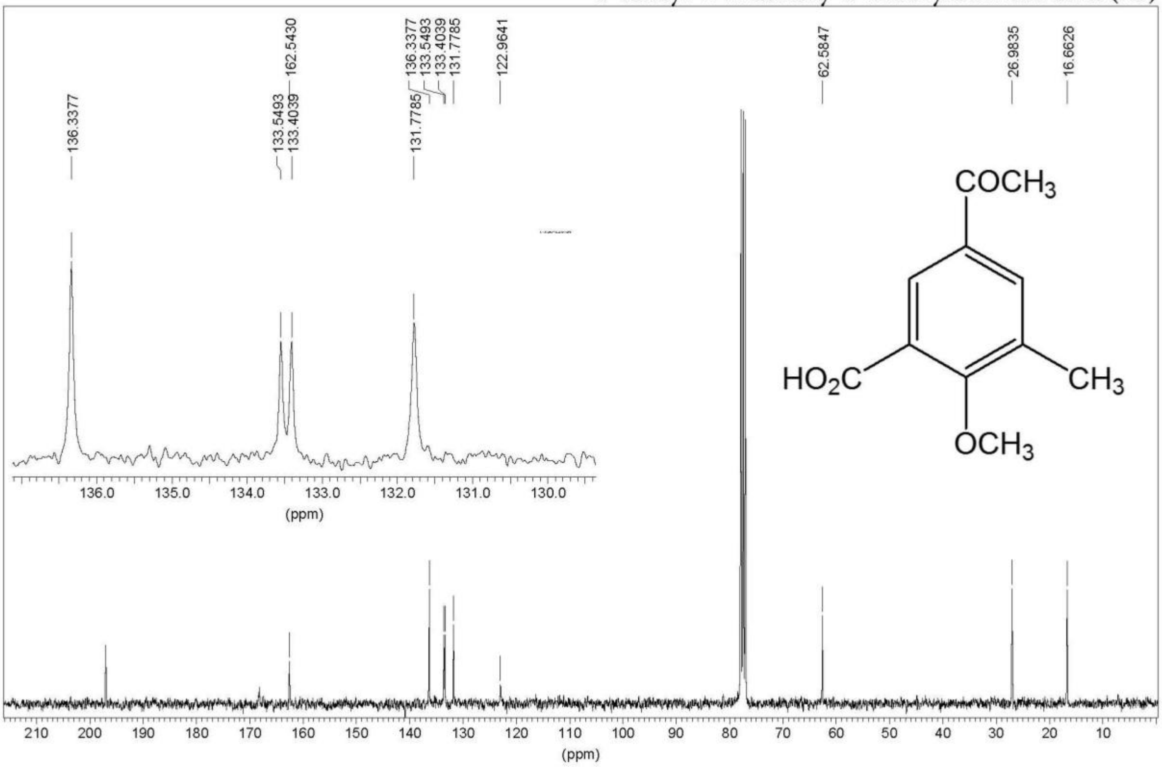

3. Computational Details

3.1. Energy Profiles for the Dissociation of Chlorides

Figure S2. Relaxed potential energy diagram (employing optimised geometry at each point) for the dissociation of chlorides anions from (**a**) radical anion of compound **12**; (**b**) radical anion of compound **5**; (**c**) radical anion of compound **13**.

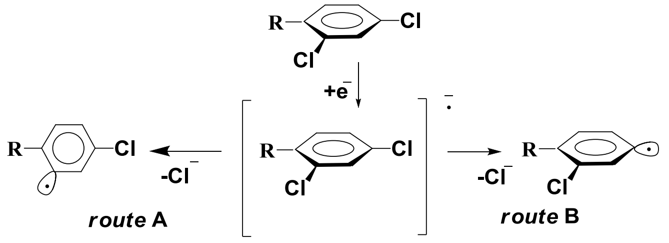

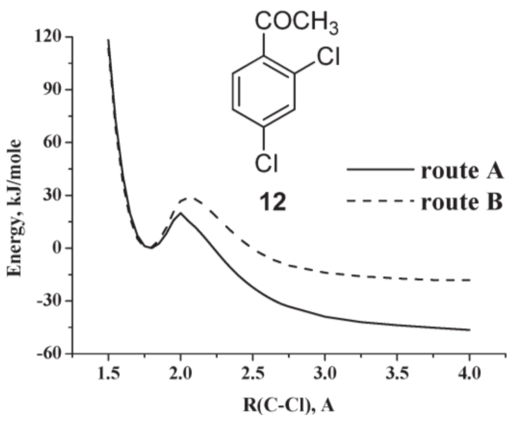

(**a**)

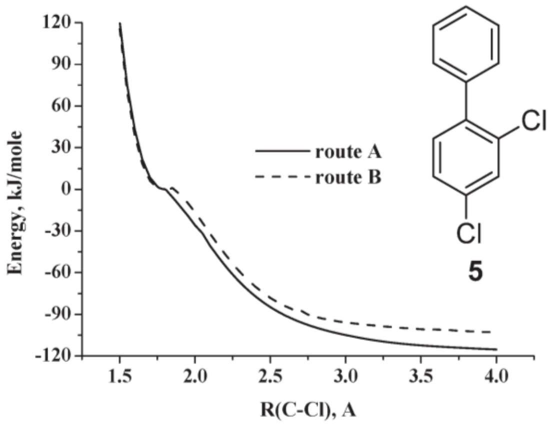

(**b**)

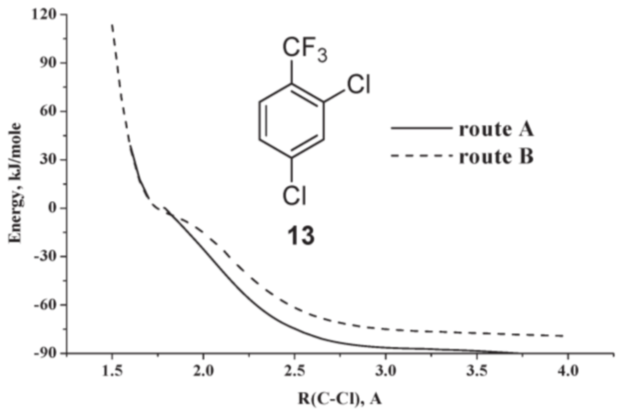

(**c**)

Table S1. Relaxed based potential energy scans (full energy optimization at each step) for the substrate **12** as a function of the C-Cl bond distance in steps of 0.05 Å.

**r(C-Cl), Å**	**E_{tot}, kJ/mol**	
	ortho	***para***
1.65	20.1	17.1
1.7	7.5	5.5
1.75	1.3	**0.5**
1.8	**0.1**	0.6
1.85	2.8	4.6
1.9	8.3	11.6
1.95	15.9	20.6
2	**18.4**	27
2.05	16	28.2
2.1	12.3	**29**
2.15	7.9	25.6
2.2	3.1	22.1
2.25	−1.9	18.1
2.3	−6.7	14
2.35	−11.1	10.1
2.4	−15.1	6.5
2.45	−18.7	3.2
2.5	−22	0.3
2.55	−24.7	−2.3
2.6	−27.3	−4.5
2.65	−29.4	−7.5
2.7	−31.3	−8.1
2.75	−32.9	−9.7
2.8	−34.7	−10.9
2.85	−36	−11.9
2.9	−37.1	−12.8
2.95	−38.1	−13.5
3	−39	−14.1
3.05	−39.7	−14.6
3.1	−40.4	−15
3.15	−42.1	−15.3
3.2	−42.5	−15.6
3.25	−42.8	−15.9
3.3	−43.2	−16.2
3.35	−43.5	−16.5
3.4	−43.8	−16.8
3.45	−44.1	−17
3.5	−44.4	−17.3
3.55	−44.7	−17.3
3.6	−45	−17.5
3.65	−45.3	−17.7
3.7	−45.5	−18.2

Table S1. *Cont.*

r(C-Cl), Å	E_{tot}, kJ/mol	
	ortho	*para*
3.75	−45.8	−18.4
3.8	−46.1	−18.7
3.85	−46.3	−18.5
3.9	−46.6	−18.7
3.95	−46.8	−19
4	−47	−19.2
4.05	−47.2	−19.4
4.1	−47.4	−19.6
4.15	−47.7	−19.8
4.2	−47.9	−20
4.25	−48.1	−20.1
4.3	−48.6	−20.4
4.35	−48.8	−20.6
4.4	−48.9	−20.8
4.45	--	−21
4.5	--	−20.3

3.2. Coordinates of All Stationary Points.

RA

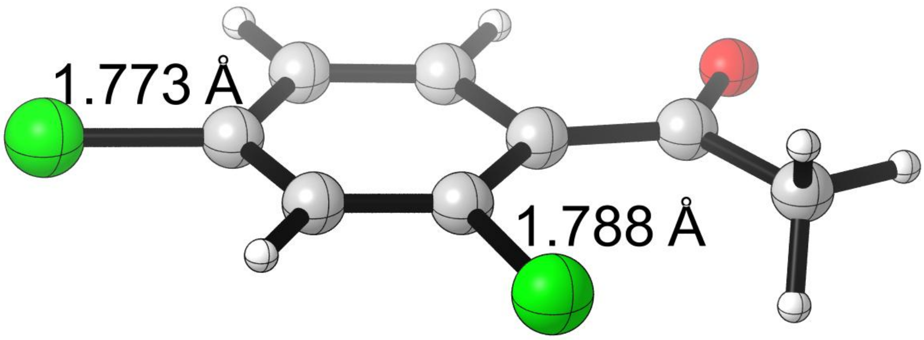

PCM Energy = −1304.19873126
C 0.636166000 1.294978000 0.013632000
C 2.012395000 1.474384000 0.038863000
C 2.859333000 0.357689000 0.037441000
C 2.317198000 −0.938448000 −0.025042000
C 0.944942000 −1.094357000 −0.051002000
C 0.000000000 0.000000000 0.000000000
Cl 4.616294000 0.592715000 0.079196000
C -1.399252000 −0.340404000 0.032087000
C -2.510674000 0.669672000 0.293739000
Cl -0.295924000 2.816227000 −0.100040000
O -1.769951000 −1.565744000 −0.128454000
H 2.427394000 2.479858000 0.040443000

H 2.970309000 −1.809644000 −0.048720000
H 0.523815000 −2.094371000 −0.093917000
H −2.284359000 1.362391000 1.110790000
H −3.410083000 0.102454000 0.555151000
H −2.744709000 1.280294000 −0.589124000

TS_{ortho}

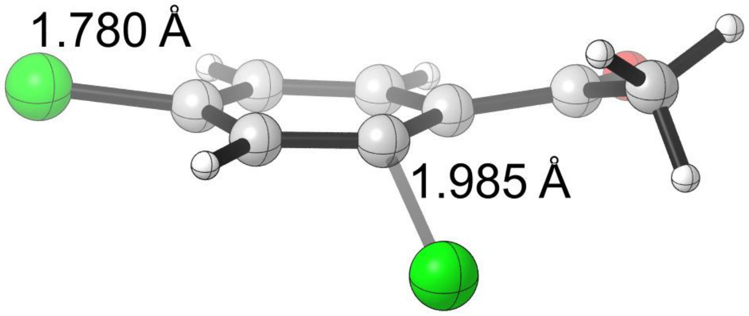

PCM Energy = −1304.19163946
C −0.307910 0.673460 0.250931
C 1.085933 0.875666 0.265156
C 1.920420 −0.198554 0.022143
C 1.420519 −1.464402 −0.377245
C 0.052827 −1.642348 −0.423841
C −0.865564 −0.617901 −0.035485
Cl 3.677831 −0.008049 0.228468
C −2.265546 −0.968876 0.135702
C −3.213447 0.009856 0.805121
Cl −1.208339 2.294766 −0.456754
O −2.725394 −2.078923 −0.243998
H 1.497091 1.865835 0.459316
H 2.104030 −2.268634 −0.640847
H −0.353657 −2.604545 −0.731723
H −2.701893 0.652395 1.528622
H −4.007686 −0.557380 1.301371
H −3.679494 0.668120 0.061235

TS$_{para}$

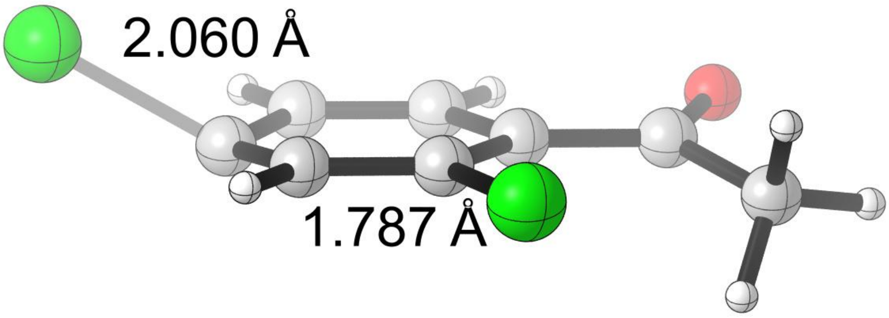

PCM Energy = −1304.18769149
C 0.281180 0.717866 0.100585
C −1.078917 0.905002 0.286738
C −1.920320 −0.208850 0.480014
C −1.389340 −1.510168 0.339997
C −0.031523 −1.667920 0.153483
C 0.888081 −0.574496 0.036164
Cl −3.809647 0.027309 −0.306289
C 2.301902 −0.930236 −0.115575
C 3.435125 0.071542 0.041722
Cl 1.223065 2.227623 −0.060663
O 2.623286 -2.120183 −0.350549
H −1.483412 1.915540 0.312895
H −2.032869 −2.384713 0.435118
H 0.395192 −2.668261 0.135797
H 3.503221 0.739844 −0.824694
H 4.367893 −0.494227 0.117212
H 3.318437 0.702995 0.927476

R_{ortho}

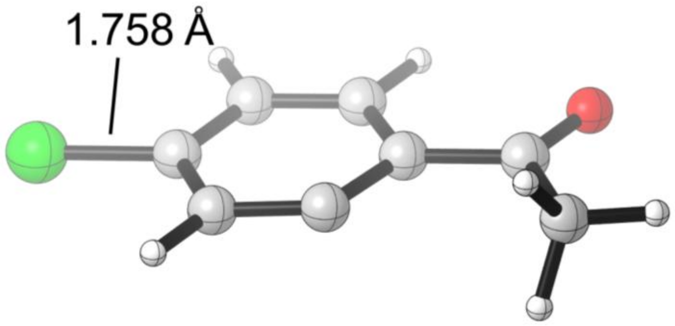

PCM Energy = −843.831901204
C −0.392165000 −1.108304000 −0.000154000
C 0.975628000 −1.243577000 −0.000208000
C 1.691317000 −0.035075000 0.000023000
C 1.043457000 1.207373000 0.000326000
C −0.348711000 1.256736000 0.000361000
C −1.116440000 0.070810000 0.000061000
Cl 3.448589000 −0.085106000 −0.000077000
C −2.609805000 0.116837000 −0.000126000
C −3.350771000 −1.200279000 0.000394000
O -3.206536000 1.191042000 −0.000631000
H 1.483301000 −2.207946000 −0.000441000
H 1.627258000 2.125994000 0.000491000
H −0.861778000 2.218091000 0.000538000
H −3.074276000 −1.792487000 −0.881148000
H −4.428301000 −1.021025000 −0.000129000
H -3.074987000 −1.791277000 0.882978000

R$_{para}$

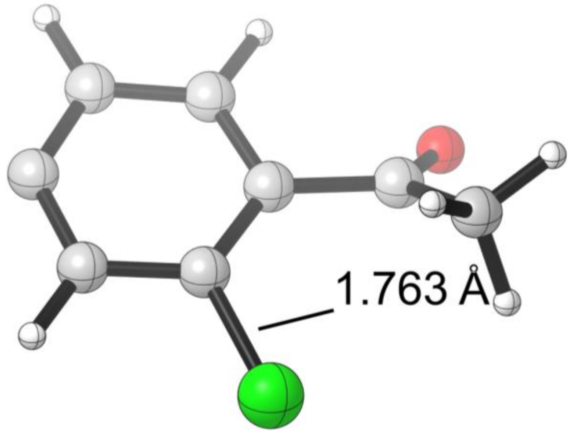

PCM Energy = −843.820708656
C 1.867333000 1.042196000 0.040390000
C 2.702037000 −0.047763000 0.132705000
C 2.321796000 −1.372594000 0.145260000
C 0.944938000 −1.612290000 0.059084000
C 0.009394000 −0.559669000 −0.010296000
C 0.493066000 0.763131000 −0.023819000
C −1.439967000 −0.968626000 −0.057911000
O −1.751877000 −1.970685000 −0.695662000
Cl −0.586148000 2.145097000 −0.209635000
C −2.490237000 −0.216524000 0.731954000
H 2.229871000 2.069246000 0.006275000
H 3.031835000 −2.195866000 0.217731000
H 0.574646000 −2.636664000 0.062585000
H −2.071058000 0.344427000 1.570980000
H −3.226854000 −0.941182000 1.092570000
H −3.009063000 0.491716000 0.074757000

© 2014 by the authors; licensee MDPI, Basel, Switzerland. This article is an open access article distributed under the terms and conditions of the Creative Commons Attribution license (http://creativecommons.org/licenses/by/3.0/).
